# Thermophilisation of communities differs between land plant lineages, land use types and elevation

**DOI:** 10.1038/s41598-023-38195-6

**Published:** 2023-07-14

**Authors:** Thomas Kiebacher, Markus Meier, Tabea Kipfer, Tobias Roth

**Affiliations:** 1 Department of Botany, Stuttgart State Museum of Natural History, Rosenstein 1, 70191 Stuttgart, Germany; 2 Department of Systematic and Evolutionary Botany, University of Zurich, Zollikerstrasse 107, 8008 Zurich, Switzerland; 3 Hintermann & Weber AG, Austrasse 2a, 4153 Reinach, Switzerland; 4 Zoological Institute, University of Basel, Basel, Switzerland

**Keywords:** Ecology, Plant sciences, Biodiversity, Climate-change ecology

## Abstract

Bryophytes provide key ecosystem services at the global scale such as carbon storage and primary production in resource limited habitats, but compared to vascular plants knowledge on how these organisms face recent climate warming is fragmentary. This is particularly critical because bryophytes differ fundamentally from vascular plants in their ecophysiological and biological characteristics, so that community alterations most likely have different dynamics. In a comparative approach, we analysed thermophilisation of bryophyte and vascular plant communities in 1146 permanent plots distributed along an elevational gradient of nearly 3.000 m in Switzerland (Central Europe) that were visited in 5-years intervals between 2001 and 2021. We estimated thermophilisation from changes in unweighted mean temperature indicator values of species, compared it to expected thermophilisation rates given the shift of isotherms and addressed differences between the two lineages, major land use types (managed grasslands, forests, unmanaged open areas), life strategy types (long- and short-lived species) and in elevation. Thermophilisation of bryophyte communities was on average 2.1 times higher than of vascular plant communities and at high elevations it approximated the expected rate given the shift of isotherms. Thermophilisation of both, bryophyte and vascular plant communities was not driven by a loss of cryophilic species but by an increase in thermophilic and mesophilic species, indicating an in-filling process. Furthermore, our data show that thermophilisation is higher in managed grasslands than in forests. We suggest that the higher responsiveness of bryophytes compared to vascular plants depends on their poikilohydry and dispersal capacity and that lower thermophilisation of forests communities is related to the buffering effect of microclimatic conditions in the interior of forests. Our study emphasises the heterogeneity of climate warming effects on plants because response dynamics differ between taxonomic groups as well as between land use types and along elevational gradients.

## Introduction

Climate warming is one of the major challenges that organisms have to face and one of the major threats to biodiversity in the twenty-first century^1–3^. Changes in species ranges, abundance and phenology successively alter extant ecosystems^4^. Spatial range shifts, poleward in latitude and upward in elevation, are among the best documented phenomena of climate warming leading to the replacement of cold-dwelling with warm-dwelling species in extant communities, i.e., thermophilisation^5^, which ultimately leads to the extinction of some of the species^6–8^.

However, range dynamics, or more specifically, how fast species track shifting isotherms, varies considerably between taxonomic groups and life strategy types^2,9–12^. Especially species' life strategies are suggested to reflect their potential to shift their ranges. For example, range shifts are usually larger and less delayed for species with high dispersal capacities and short life cycles^11,13,14^.

Furthermore, it has been demonstrated that disturbance accelerates thermophilisation, because it determines species turnover and facilitates the establishment of species better adapted to elevated temperatures^15–18^. Consequently, plant communities subjected to different disturbance regimes due to different land use can be expected to differ in the magnitude of thermophilisation. However, this has rarely been addressed using the same sampling strategy for different land use types, because monitoring programs and historical surveys available for resampling are often restricted to one land use type, typically forests or grasslands. Thermophilisation in managed grassland that are regularly disturbed through mowing or grazing may be higher than in unmanaged habitats or forests characterised by rare disturbance through logging.

For plants, substantial time lags were observed with realised range shifts being much smaller than expected given the observed temperature increase, both at the community and the species level^12,18–21^. But whether time lags differ along elevational gradient is still poorly understood. Thermophilisation of forest communities was found to increase with elevation^19,22^ while range shifts of species were observed to decrease with elevation^23,24^.

Studies of the impacts of climate warming on plants are highly biased towards vascular plants. Bryophytes, the second group of land plants, hitherto received little attention^20,25^, although they often constitute a large part of the local and regional plant diversity and provide key ecosystem services^26,27^. Bryophytes play a significant role at the global scale in carbon storage, nutrient and water cycling and the resilience of ecosystems (e.g., Turetsky et al.^28^). Compared to vascular plants, they are generally more diverse and abundant in harsh environments, e.g., at high latitudes and elevations^29–31^ and are considered particularly threatened by climate warming^25,32^. Even less attention than to bryophytes as an entire group was paid to life strategy types among them. They encompass a wide range, from fugitive and very short-lived (a few weeks) types inhabiting transient (micro-)habitats, to competitive and long-lived types that dominate persistent communities.

The knowledge gap in bryophytes is particularly critical because they fundamentally differ from vascular plants in their ecophysiological properties and are thus expected to be differently affected by climate warming^25,33^. They are poikilohydric (i.e., they lack the ability to regulate the water content) and they absorb humidity and nutrients directly from the atmosphere. Hence, they are more directly linked to the abiotic environment and are usually more sensitive to environmental changes^27,34,35^. Furthermore, many bryophyte species do not depend on a well-developed soil layer and some of them can grow directly on bare rock surfaces. This makes them excellent pioneer species, able to colonise habitats where competing vascular plants are absent. Finally, bryophytes differ from vascular plants in their high dispersal capacity^36^. Most species are dispersed by wind via small diaspores (usually spores in the magnitude of 10–30 µm) while diaspores of vascular plants are generally much larger, and this difference shapes their distribution patterns^37^.

Here, we use a comparative approach to assess the impact of contemporary climate warming on bryophytes and vascular plants in a mountainous region in Central Europe. We examined data from 1146 permanent plots spanning an elevational gradient of nearly 3000 m that were surveyed 4 to 5 times each between 2001 and 2021 in the frame of the Biodiversity Monitoring Switzerland (https://www.biodiversitymonitoring.ch). This monitoring programme surveys bryophytes and vascular plants on the same study plots using standardised schemes and provides a unique opportunity to infer how climate warming affects the two lineages. We tested whether: (i) Temperature affinities of communities changed over time, if the changes differed along the elevational gradient and whether they were driven by an increase or decrease of cryophilic, mesophilic or thermophilic species, respectively. (ii) Thermophilisation is more pronounced among bryophyte than among vascular plant communities as anticipated because of their ecophysiological properties and higher dispersal capacities. (iii) The magnitude of climate warming effects differs between major land use types. We expected effects in managed grasslands to be higher than in forests and unmanaged open areas comprising mostly unmanaged grasslands above the tree line, scree and rocks. (iv) Species with a short-lived life strategy respond more strongly to climate warming than species with a long-lived strategy.

## Material and methods

### Study design and sampling

Field data are from the Biodiversity Monitoring Switzerland^38^ (BDM, https://www.biodiversitymonitoring.ch) and consists of a total of 1446 circular 10 m^2^ permanent plots on a regular grid laid out over entire Switzerland (Supplementary Fig. S1 online). The county is situated in the temperate climate zone, covers an area of c. 40,000 km^2^ and consists of three main physiographic regions: The Jura Mountains in the Northwest (up to c. 1700 m a.s.l. high), the Central Plateau (around 400–600 m) and the Swiss Alps with the highest peaks exceeding 4500 m in elevation. Mean annual temperatures range from below -5 degrees at the highest elevations to around 12° in the lowlands and precipitations range from ca. 500 to more than 2000 mm per year. In each plot the presence of bryophyte and vascular plants species is recorded in 5 years intervals since 2001. Vascular plant species are recorded in the field and small parts of bryophyte species growing up to 1.5 m above ground are collected from the wild and identified microscopically by specialists in the lab (Supplementary Tables S1 and S2 online). In order to minimize sampling bias of bryophytes among the non-specialist collectors (ca. 20 per survey) all substrate types (e.g., deadwood, soil) are sampled independently and each plot is screened in concentric circles to guarantee that the whole surface is examined^39,40^. The collectors are regularly trained to follow the method meticulously and the sampling biases between the collectors and an independent controller who re-samples a random subset of the plots was constant over the years. Also, all other procedures of the monitoring program, including species determination are carried out following standardised protocols and are subjected to different steps of quality control^39^. Voucher information including the name of the determinator of each specimen is stored and available at data base of the Swiss National Data Centre for Bryophytes (https://www.swissbryophytes.ch). Field work and the collection of bryophyte material are carried out in accordance with institutional, national and international guidelines and legislation and permission is provided by the Swiss Federal Office for the Environment.

We analysed the data from 2001 to 2021 and selected the plots where each survey was consistently assigned to one of the three major land use types managed grasslands, forests or unmanaged open areas. The latter comprise alpine grasslands, pioneer vegetation, scree and rock faces, alpine flood plains and snowbeds. A total of 1146 plots met the selection criteria (Supplementary Fig. S1 online), covering an elevational range from 268 to 3060 m a.s.l. In order to consider thermophilisation at different elevational zones separately, we assigned each plot to one of the zones proposed by Schreiber et al.^41^ according to the temperature zonation in Switzerland: colline, montane, subalpine and alpine (Supplementary Table S3 online). Generally, the zones extend higher up in the Southern and in the Central Alps than in the Northern Alps and the Jura Mountains. Roughly, the colline zone ranges to 600–900, the montane zone to 1200–1700, the subalpine zone to 1900–2400 and the alpine zone to 2700–3100 m a.s.l. Note, that we pooled eight plots in the nival zone with those in the alpine zone. On average, each plot was sampled 4.1 ± 0.4 SD times and across all 4710 surveys, 546 bryophyte and 1244 vascular plant species were recorded (Supplementary Tables S1 and S2 online). The mean number of species per survey was 12.9 ± 8.8 for bryophytes and 27.3 ± 15.1 for vascular plants (Supplementary Table S4 online).

### Community temperature index (CTI), thermophilisation rate and notional elevation shift (NES)

To investigate thermophilisation processes in plant communities we used the per plot temporal change in temperature affinities of the bryophyte and vascular plant communities. To this end we first calculated the per survey community temperature index (CTI) of each community (lineage per plot per survey) as the unweighted mean of the Landolt temperature values^42^ of recorded species. Similar to Ellenberg indicator values^43^, the Landolt values are ordinal numbers ranging from 1 (most cryophilic) to 5 (most thermophilic) and express the realised ecological optima of species. They were developed for the specific situation in Switzerland. To identify which groups of species caused thermophilisation of communities we examined the temporal change in the number of three categories of species with different temperature affinities. With respect to the temperature range in Switzerland we termed species with Landolt temperature values of 1–2.5, 3, 3.5–5 as cryophilic, mesophilic and thermophilic species, respectively. The Landolt temperature values were available for 74% of recorded bryophyte species (81% of individual observations) and for 96% of recorded vascular plant species (87%). Missing values are mostly due to species for which Landolt values are not defined because their temperature affinity spans more than three ranks^42^ (Supplementary Tables S1 and S2 online).

From CTIs we then calculated the per plot thermophilisation rate using linear models (LMs) and year of survey as predictor variable. The thermophilisation rate is thus the average change in CTI per year. In this way we obtained thermophilisation rates for a total of 1057 bryophyte and 1138 vascular plant communities. The difference of these numbers with respect to the total number of plots of 1146 is explained by the plots for which CTI could not be calculated in at least two surveys because Landolt temperature values were not available for the species present in the respective survey (usually species-poor plots).

From the thermophilisation rate of each community we then calculated its notional elevation shift (NES) which is the shift in elevation in meters per decade, that corresponds to the observed change in CTI with respect to the average change in CTI along the elevational gradient. In other words, it is the difference in elevation at which communities with the same CTI are observed after 10 years. For example, if the thermophilisation rate per year is 0.01 and the average change in CTI along the elevational is 0.1 per 100 m, the NES would equal 100 m per decade. We introduced this measure to (a) correct for the difference in the average change in CTIs of bryophyte and vascular plant communities along the elevational gradient (see below) and hence to allow us to compare the two groups; (b) to compare the observed thermophilisation with expected values given the observed temperature increase; and c) to relate our results to thermophilisation rates reported in other studies. To estimate the NES of plant communities we first applied LMs separately for bryophytes and vascular plants to calculate the average decrease of CTI across elevation using the mean CTI of all surveys of each plot as dependent variable (bryophytes: −0.082 per 100 m, R^2^ = 0.67, *p* < 0.001; vascular plants: −0.098 per 100 m, R^2^ = 0.89, *p* < 0.001). Then, we calculated the NES of each bryophyte and vascular plant community by dividing its thermophilisation rate by the average decreases of the CTI across elevation, i.e., by 0.082 for bryophytes and 0.098 for vascular plants. By multiplying with 1000 we got the NES in meters per decade which we use as unit throughout the study. We provide mean values of NES ± SD per lineage, land use type and elevational zone in Supplementary Table S3 online.

### Shift of isotherms

To relate the thermophilisation of communities to the observed temperature increase we approximated the elevation shift of isotherms as follows. We first calculated a warming of 0.42 °C per decade from mean annual temperatures in Switzerland between 2001 and 2021^44^ using a LM (R^2^ = 0.2164, *p* = 0.034). This value is largely consistent with other estimates for the study region considering similar time periods^45,46^ and assuming a temperature decrease with elevation (i.e., the adiabatic lapse rate) of 0.5 to 0.67 °C per 100 m in Switzerland^46^ it corresponds to an upward shift of isotherms of 63 to 84 m per decade.

### Life strategies

We compared NESs of communities of species with a short-lived life strategy with those of species with a long-lived strategy. To assign bryophyte species to these two strategy types we used the system proposed by During^47,48^ and the species' classification of Dierssen^49^ with few edits and additions for species not covered based on our expertise on the species in Switzerland (Supplementary Table S1 online). We summarised fugitives, annual shuttles (defined as annual species adapted to microhabitats that disappear predictably at varying rates but reappear frequently within the same community), colonists (species with a potential life span of few years and adapted to colonize open habitats; including ephemeral colonists and pioneer colonists) and short-lived shuttles (shuttle species with a life span of a few years) as short-lived life strategy, and long-lived shuttles, perennials, competitive perennials, stress tolerant perennials and dominants (all defined as species with a life span of many years) as long-lived life strategy. Similarly, for vascular plants we used the life strategy classification proposed by Landolt et al.^42^ and classified predominantly ruderal strategists (attributes: rrr, rrs and crr) as short-lived species and predominantly competitive strategists (attributes: ccc, ccs and ccr) as long-lived species (Supplementary Table S2 online). Predominantly stress-tolerant strategists (attributes: sss, css and rss) and competitive-ruderal-stress-tolerant strategists (attribute: crs) were excluded from the analysis because longevity is not defined for the stress-tolerant category^42^.

### Statistical analysis

#### CTI models

To test if CTI changed over time within lineages and land use types at each elevational zone, we applied linear mixed models (LMMs) with CTI as dependent variable, year of survey as predictor variable and plot ID as random intercept variable. We applied separate models for each factor combination of lineage (bryophytes, vascular plants), land use type (managed grasslands, forests, unmanaged open areas) and elevational zone (colline, montane, subalpine, alpine) except for unmanaged open areas in the colline, montane and subalpine zone and forests in the alpine zone due to low representation (< 14 plots per factor combination; Supplementary Table S3 online). Hence, in total we run 16 models (2 plant lineages × 4 elevational zones × 2 land use types per elevational zone) with the formula: *CTI* ~ *year of survey* + *plot ID *(*random effect*) and calculated 95% confidence intervals based on 1000 bootstrap samples.

#### NES models

We then proceeded to analyse the per plot NES to test for differences between thermophilisation of bryophyte and vascular plant communities, land use types and elevation by applying LMMs to the full data set (Supplementary Table S3 online). To test and quantify the average difference between the two lineages we first run a LMM using solely lineage (0: bryophytes; 1: vascular plants) as predictor variable and plot ID as random intercept variable: NES lineage model; *NES* ~ *plant lineage* + *plot ID *(*random effect*). To account for decreased residual variation with increasing number of species we used a power variance function^50^.

Then, we constructed a more complex model by adding land use type (0: managed grasslands; 1: forests; 2: unmanaged open areas) and elevation (numeric variable) and all two-way and three-way interactions as predictors to the NES lineage model: NES full model; *NES* ~ *plant lineage* + *elevation* + *land use type* + *plant lineage *×* elevation* + *plant lineage *×* land use type* + *elevation *×* land use type* + *plant lineage *×* elevation *×* land use type* + *plot ID *(*random effect*)*.* To obtain interpretable estimates we z-transformed elevation by subtracting 514 m (i.e., the mean elevation of the colline plots; Supplementary Table S3 online) and dividing it by 100 m. The estimate of the intercept is thus an approximation of the NES in the colline zone (the NES at 514 m a.s.l.) and the estimate of the elevation effect is the change of the NES per 100 m increase in elevation. To derive parameter estimates we rerun the model without non-significant interactions.

#### Number of species models

To estimate the temporal change in the number of species of three species groups with different temperature affinity (cryophilic, mesophilic and thermophilic species) we applied generalized LMMs with a Poisson error structure and a log-link function. We specified the number of species recorded during a survey as dependent variable, year of the survey as predictor variable and plot ID as random intercept variable. Because we were interested in changes within the two lineages and because we expected differences along the elevational gradient, we applied separate models for each plant lineage and species group across all elevational zones (2 plant lineages × 3 species groups = 6 models) and then also distinguished between the 4 elevational zones (3 species groups × 2 plant lineages × 4 elevational zones = 24 models). The model formula for these models was*: Number of species* ~ *year of survey* + *plot ID *(*random effect*)*.*

#### Life strategy models

To infer whether NES differed between short-lived and long-lived species we applied LMMs using NES as dependent variable, life strategies (0: short-lived species; 1: long-lived species), elevation (numeric variable), and land use type (0: managed grasslands; 1: forests; 2: unmanaged open areas) as predictor variables, and plot ID as a random intercept variable. Again, we applied a power variance function^50^. We run separate models for bryophytes and vascular plants with the formula *NES* ~ *life strategy* + *elevation* + *land use type* + *plot ID *(*random effect*)*.*

All analyses were run in R (version 4.1.0)^51^. We used the gls and lme function of the R-package nlme (version 3.1-152; Pinheiro et al. 2021) to analyse CTI and NES and the glmer function of the lme4 package (version 1.1-27)^52^ to infer the temporal change in numbers of species. Unless otherwise specified above, we used default settings. For all models we examined model assumptions (normality, homoscedasticity, no spatial patterns of residuals) using residual analyses.

## Results

### Thermophilisation, elevation and number of species

**Figure 1:**
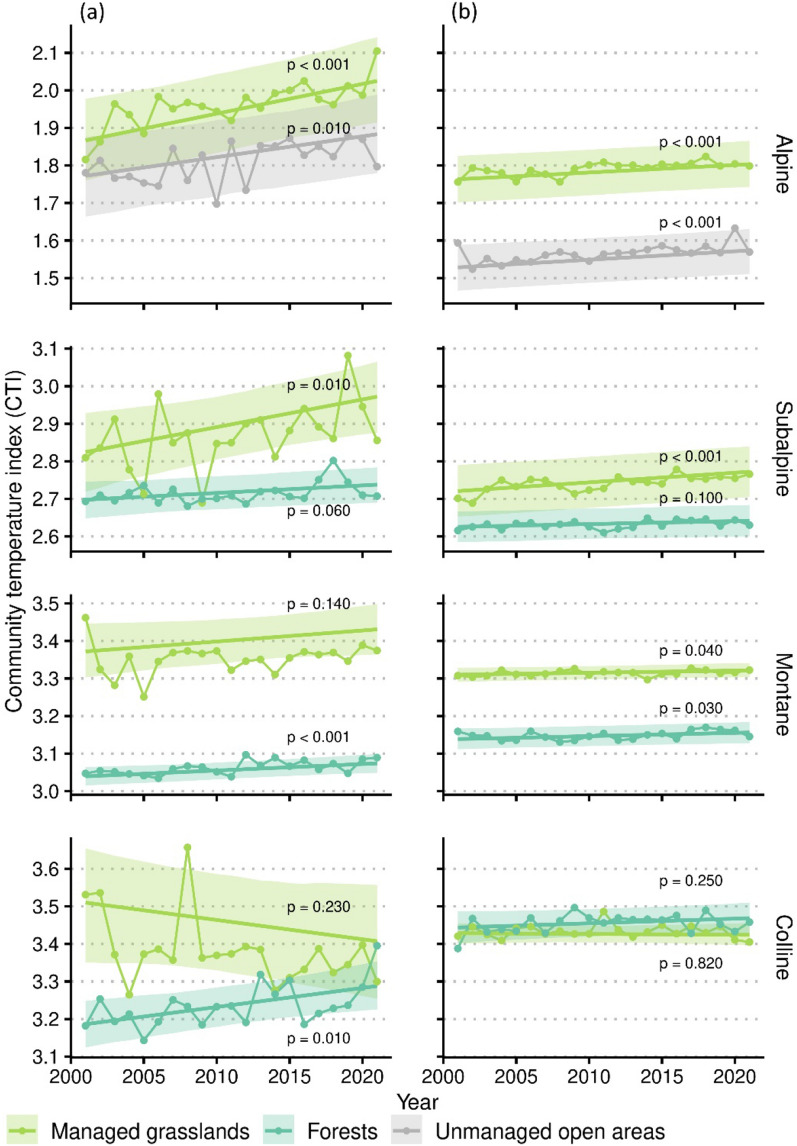
Change in community temperature index (CTI) in Switzerland between 2001 and 2021 of bryophytes (**a**) and vascular plants (**b**) in three major land use types and in four elevational zones (CTI models). Solid lines are estimates with 95% confidence intervals (shaded areas) with *p-*values of the temporal change in CTI, data points are median CTIs per year.

**Figure 2:**
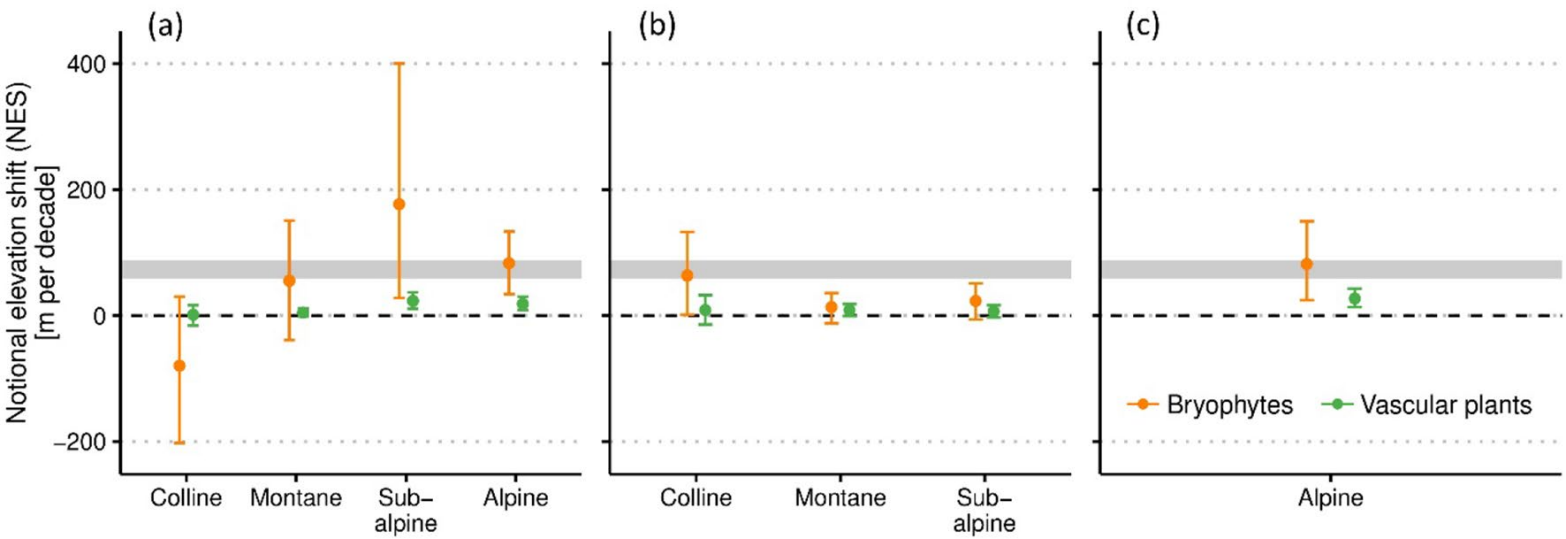
Mean notional elevation shifts (NES) of bryophyte and vascular plant communities in managed grasslands (**a**), forests (**b**) and unmanaged open areas (**c**) in Switzerland between 2001 and 2021. The whiskers are bootstrapped 95% confidence intervals. The grey bar marks the observed upward shift of isotherms of 63 to 84 m per decade.

We observed thermophilisation of communities across all elevational zones, land use types and in both lineages (CTI models; Figs. 1 and 2). The CTI increased in almost all factor combinations of plant lineage (bryophytes, vascular plants), elevational zone (colline, montane, subalpine, alpine) and land use type (managed grasslands, forests, unmanaged open areas) and in most factor combinations the increase was supported at the *p* < 0.05 level. While in the alpine zone we found a significant (*p* ≤ 0.01) increase of the CTI of bryophytes and vascular plants in both major land use types assessed in this elevational zone (managed grasslands, unmanaged open areas), the increase in CTI was generally lower and less supported at lower elevations. Accordingly, we detected a significant effect of elevation in the NES full model (*p* = 0.005, after removing non-significant interactions; Table 1) with the NES increasing by 1.1 m per 100 m increase in elevation. At the colline zone we detected a significant increase of the CTI only for bryophytes in forests.

**Table 1:** Variation of the notional elevation shift (NES) of communities between plant lineages, along the elevation gradient and between land use types.

Predictor	DF	F-value	*p*
(a) ANOVA table of full model			
Plant lineage	1	11.05	< 0.001
Elevation (per 100 m)	1	9.75	0.002
Land use type	2	2.96	0.052
Plant lineage × elevation	1	2.08	0.150
Plant lineage × land use type	2	1.71	0.182
Elevation × land use type	2	2.34	0.097
Plant lineage × land use type × elevation	2	1.16	0.313

Parameter	Estimate	SE	*p*
(b) Parameter estimates of full model			
Intercept	16.09	23.39	0.492
Vascular plants	−10.98	23.77	0.644
Elevation	2.92	1.64	0.074
Land use type: forests	1.51	26.13	0.954
Land use type: unmanaged open areas	−87.67	55.47	0.114
Vascular plants × elevation	−1.66	1.68	0.325
Vascular plants × forests	5.55	27.61	0.841
Vascular plants × unmanaged open areas	106.74	62.77	0.089
Elevation × forests	−2.76	2.15	0.200
Elevation × unmanaged open areas	3.06	3.33	0.358
Vascular plants × elevation × forests	0.91	2.39	0.702
Vascular plants × elevation × unmanaged open areas	−4.82	3.77	0.201
(c) Parameter estimates with non-significant interactions removed			
NES of bryophytes in managed grasslands at 514 m a.s.l. (intercept)	26.35	6.85	< 0.001
Difference between vascular plants and bryophytes	−18.26	5.02	< 0.001
Elevation (per 100 m)	1.10	0.40	0.005
Land use type: forests	−10.39	4.92	0.035
Land use type: unmanaged open areas	−12.00	7.50	0.110

Table (a) reports the ANOVA table of the NES full model, table (b) the parameter estimates and table (c) the parameter estimates after removing non-significant interactions.

At all elevational zones the number of cryophilic, mesophilic and thermophilic bryophyte and vascular plants species either increased or showed no significant change (number of species models; Table 2). Mesophilic and/or thermophilic species of both lineages increased at all elevational zones except for vascular plants in the colline zone. Cryophilic species did not show a significant change except for an increase in vascular plants in the alpine zone and across the entire gradient.

**Table 2:** Change in the number of cryo-, meso- and thermophilic bryophyte and vascular plant species in colline, montane, subalpine and alpine plant communities in Switzerland between 2001 and 2021 (number of species models).

	Bryophytes			Vascular plants		
	Estimates	SE	*p*	Estimates	SE	*p*
Colline						
Cryophilic species	−0.092	0.216	0.671	−0.007	0.257	0.979
Mesophilic species	**0.095**	**0.038**	**0.012**	0.030	0.031	0.333
Thermophilic species	**0.262**	**0.001**	** < 0.001**	0.047	0.035	0.176
Montane						
Cryophilic species	0.012	0.062	0.846	−0.069	0.050	0.171
Mesophilic species	**0.037**	**0.015**	**0.010**	0.010	0.012	0.429
Thermophilic species	**0.198**	**0.041**	** < 0.001**	**0.057**	**0.023**	**0.016**
Subalpine						
Cryophilic species	0.015	0.032	0.633	−0.028	0.020	0.156
Mesophilic species	**0.047**	**0.018**	**0.009**	**0.049**	**0.017**	**0.003**
Thermophilic species	0.140	0.098	0.152	0.046	0.053	0.383
Alpine						
Cryophilic species	0.025	0.021	0.248	**0.051**	**0.011**	** < 0.001**
Mesophilic species	**0.091**	**0.028**	**0.001**	**0.090**	**0.036**	**0.012**
Thermophilic species	0.105	0.119	0.377	**0.245**	**0.001**	** < 0.001**
Entire elevational gradient						
Cryophilic species	0.022	0.017	0.206	**0.028**	**0.010**	**0.004**
Mesophilic species	**0.051**	**0.010**	** < 0.001**	**0.028**	**0.009**	**0.002**
Thermophilic species	**0.198**	**0.032**	** < 0.001**	**0.057**	**0.018**	**0.001**

Trends significant at the *p* ≤ 0.05 level are displayed in bold.

### Bryophytes vs. vascular plants

The NES of bryophyte communities was on average 2.1 times higher than of vascular plant communities (NES lineage model: NES of bryophytes [intercept] = 29.8 ± 4.3 SE, *t* = 6.9; *p* < 0.001, estimate plant lineage [vascular plants] = -15.8 ± 4.8, *t* = 4.8, *p* = 0.001). In the subalpine and alpine zone, the NES of bryophytes averaged 78 and 76 m per decade while vascular plant communities notionally migrated upwards by 15 and 25 m on average in these zones, respectively (Supplementary Table S3 online).

### Land use types and life strategies

The LMM analysis of the whole data set (NES full model) revealed a marginally significant effect (*p* = 0.052) of land use type on the NES of communities, and, after removing non-significant interactions significantly higher shifts in managed grasslands compared to forests (Table 1a − c). The NES per decade in managed grasslands was about 10 m higher than in forests while the NES in unmanaged open areas was statistically not different from the NES in managed grasslands (Table 1c). Notably in the alpine zone the NES was similar in unmanaged open areas and managed grasslands (Fig. 2).

We did not detect significant differences in the NES of short-lived vs. long-lived bryophyte and vascular plant species (Fig. S2, Table S5 in Online Resource 1). For bryophytes, however, in all land use types average values were higher for short-lived species (Supplementary Fig. S2 online).

## Discussion

Here, we supplement research on climate warming effects with results based on an extensive data set from exactly relocated plots monitored for 15 to 20 years and considering both lineages of land plants, bryophytes and vascular plants. Our analyses support the hypothesis that thermophilisation (in terms of NESs) is higher in bryophyte than in vascular plant communities and in managed grasslands compared to forests. In contrast, thermophilisation in unmanaged open areas was not statistically different from managed grasslands and between short-lived and long-lived strategy types. Furthermore, our results indicate that thermophilisation increases with elevation and is due to the invasion of thermophilic and mesophilic species rather than the loss of cryophilic species.

So far, observational studies addressing climate warming effects on plants communities and species were often based on the reassessment of historical surveys (e.g., Lenoir et al.^53^, Cannone and Pignatti^54^, Rumpf et al.^24^). Compared to our study, data from these approaches have the advantage that they encompass longer time intervals and are thus less susceptible to short-term confounding effects such as single years of extreme weather conditions, and that they include the onset or earlier stages of recent climate warming. Furthermore, sampling effects that may arise from the periodical resampling of plots do not apply. On the other hand, the repetition of historical surveys is usually associated with considerable uncertainties regarding the spatial relocation^55^ and the sampling procedure (e.g., accuracy) and historical surveys that considered both, vascular plants and bryophytes are rare (see Vitasse et al. 2021^45^). Furthermore, the confounding effect of other components of global change may have been stronger in the second half of the twentieth century as compared to the last 20 years considered here. For instance, the deposition of nitrogen, which has an important effect on frost tolerance^56^ as well as on competitiveness and plant growth in general, was highest in the 1980s and has since declined in Europe including Switzerland^57,58^. Accordingly, e.g., Becker Scarpitta et al. ^35^ observed an increase in nitrogen indicator values in bryophyte communities in France in the period 1976–2012 whereas in the period 2003–2017 no such change could be detected in Switzerland^59^. Another factor that substantially affects bryophyte communities is the atmospheric sulphur dioxide load^60^ which in Europe likewise climaxed in the 1980s ^61^. It has been shown for southern Belgium, that changes in epiphytic communities are predominantly owed to improved air quality when considering the last four decades but that currently the regional climate better explains variation in species occurrences than air quality^62^. Therefore, and because the sulphur dioxide load in Switzerland was much less severe^63^ it is unlikely that changes in the sulphur dioxide load per se significantly influenced our results. However, most likely also in the time period considered here other factors than temperature influenced community composition of plants in Switzerland such as increasing CO_2_, the initiatives to promote biodiversity^64^ and improving air quality in general^57,65^.

### Thermophilisation, elevation and number of species according to temperature affinity

Although relative to many other studies (e.g., Bertrand et al.^19^, De Frenne et al.^66^, Becker Scarpitta et al.^35^) our data set comprised a rather short time interval of two decades we found clear evidence for thermophilisation of plant communities in our study system in Central Europe. Thermophilisation was higher at high elevations, but occurs along the entire elevational gradient, and in natural as well as managed habitats. Non-significant increase of CTI in some of the factor combinations of plant lineage (bryophytes, vascular plants), elevational zone (colline, montane, subalpine, alpine) and land use type (managed grasslands, forests, unmanaged open areas; Fig. 1) coincided with low representation of plots (cf. Fig. 1, Supplementary Table S3 online) and thus most likely rely on sampling effects. For instance, the CTI significantly increased in montane forests in both lineages, but only marginally so in subalpine forests where sample size was less than half. However, at low elevation thermophilisation was generally less pronounced and we could not detect any signal of thermophilisation of vascular plant communities at the colline zone. Neither CTI nor the number of thermophilic species increased, indicating that thermophilisation at low elevations is constrained.

Similarly, lower thermophilisation of vascular plant communities and smaller range shifts of species at low elevations were observed by Bertrand et al.^19,22^ and Savage and Vellend^67^, and were attributed to small and/or weakly connected local pools of thermophilic species better adapted to the new conditions. Immigration of such species along latitudinal gradients is constraint by long distances^68^ and barriers such as mountains, e.g., the Alpine arc which in Switzerland separates the northern lowlands from warmer regions in the South. Our finding that the number of meso- and thermophilic vascular plant species did not significantly increase in the colline zone supports this scenario. By contrast, the increase of these groups in bryophytes was highly significant and can be explained by their high dispersal capacities. Bryophytes have small diaspores and are easily dispersed over large distances^36^ (Medina et al. 2011). Furthermore, compared to vascular plants they are more dependent on microclimatic conditions than on macroclimate. Thermophilic species may thus more easily spread from exceptionally warm microsites whereas thermophilic vascular plants face larger immigration distances. However, Zanatta et al.^69^, using a modelling approach, suggested that the ability to track shifting isotherms is also constraint in bryophytes, especially for species with large spores. This can explain, why in our study also thermophilisation of bryophyte communities increased with elevation (non-significant interaction plant lineage × elevation). The short distances along the generally steep elevational gradient in Switzerland seem to facilitate the migration of both, vascular plants and bryophytes. Recently, it has also been shown, that intraspecific trait variability of lowland species is higher compared to highland species^70^ which could result in higher resilience of lowland species and may contributed to the positive effect of elevation.

However, smaller NESs at lower elevations inferred from our presence-absence data, hence, from abundance unweighted CTIs, should not uncritically be interpreted as lower impacts of climate warming. As has been suggested for vascular plants^67,71^, thermophilisation of communities at low elevations may in the short term be expressed more in changes in abundance than in species composition.

### Magnitude of thermophilisation

Bertrand et al.^19^ assessed thermophilistion of vascular plant communities in French forests between 1965 and 2008 and found negligible thermophilisation in lowland areas (< 500 m a.s.l.) as opposed to a rate of 50% of the expected given the observed temperature increase in highland areas (500–2600 m a.s.l.). The latter value is higher than the maximum rate detected for vascular plants in our study (30 to 40% in the alpine zone; NES of 25.0 m/observed shift of isotherms of 63 to 84 m, Supplementary Table S3 online) and may indicate that the accelerated warming of the last two decades results in larger climatic debts as compared to time intervals including the initial phase of climate warming characterised by a moderate temperature increase that allowed communities to better track climatic suitable conditions. However, the comparison of the two studies needs to be taken with caution, because Bertrand et al.^19^ used a different method to quantify thermophilisation, i.e., a modelling approach using the floristic assemblage of surveys conducted before the onset of recent climate warming (defined as before 1985) as reference. Compared to Bertrand's et al.^19^ and our occurence-based estimates of thermophilisation, abbundance weighted estimates from surveys on European mountain summits revealed higher rates that were partly close to the expected^5,72^ and suggest that increasing abbundance of mesophilic and thermophilic species is a substantial component of thermophilisation of vascular plant communities in mountain environments.

If thermophilisation of communities is interpreted as the consortium of range shifts of its species, mean range shifts of species may, with caution, be compared to our data and it must be noted that the following publications considered longer time spans: mean range shifts of vascular plants varied largely between studies, but in agreement with our results they were mostly below 50% of the expected (e.g., Chen et al.^73^, Rumpf et al.^24^, Lenoir et al.^12^). Studies on bryophytes are scarce. Bergamini et al.^74^ used herbarium specimens to estimate elevational range shifts of 61 bryophyte species in Switzerland over a time interval of 60 to 100 years (collected 1880–1920 vs. 1980–2005) and found an average shift of the mean elevation of species of 43% of the expected due to the observed temperature increase during the mid-points of the time periods. This rate is similar to the rate observed at the community level in our study (35 to 47%; NES of 29.8 m based on NES lineage model/observed shift of isotherms of 63 to 84 m) and could indicate that time lags did not increase for bryophytes although the warming accelerated in the last two decades^46^.

### Bryophytes vs. vascular plants

The NES of bryophyte communities was higher than of vascular plant communities and in the subalpine and alpine zone it approximated the upward shift of isotherms, while the NES of vascular plant communities was below half of the expected (Supplementary Table S3 online). We suggest that these patterns are mainly related to the ecophysiological and biological differences between the two lineages as pointed out in the introduction, i.e., poikilohydry of bryophytes and different life strategies. Many bryophyte species have a colonist strategy with short generation times and high reproductive effort^47–49^ which facilitates them to colonise sites that become suitable through climate warming. By contrast, mountain vascular plant species are mostly long-lived and form persistent communities which have high resilience to invasion^75,76^. Furthermore, upward range shifts of vascular plants depend on the availability of soil while the desiccation tolerance and nutrient uptake mechanisms of bryophytes make them much less dependent on a substrate that serves as water storage and source of nutrients^34^. Another factor which may favour thermophilisation of bryophyte communities, is that the distribution of bryophyte species along elevational gradients is notably modified by microsite availability, while the distribution of vascular plants is more directly linked to the gradual temperature change along elevational gradients^29,77^. Thus, invasion of thermophilic bryophyte species may often occur horizontally from warmer microsites within the same elevational band. This hypothesis is supported by results of Bergamini et al.^74^, who found no increase in elevational ranges of meso- and thermophilic bryophyte species. Horizontal migration from warmer microsites means shorter distances which in tandem with the high mobility through small diaspores dispersed by wind^78^ can facilitate invasion. However, horizontal migration has been proposed as an important factor also for invasion of vascular plants^54,79^ but may be less pronounced because of larger diaspores. Finally, vascular plant growth and expansion at high elevations is slowed down by low temperatures, short growing season and positive net photosynthesis being mostly limited to the summer season^5^. In contrast, bryophytes can maintain positive net photosynthesis rates at very low temperatures (e.g., down to −10 °C in *Pellia epiphylla*^80^, a common liverwort in the study region) and are opportunistic in their assimilation strategy. Once environmental conditions are favourable, they can reassume positive net photosynthesis within short time intervals (often within minutes) throughout the year^30,34^. The few studies that so far considered both, bryophytes and vascular plants in the context of climate warming mostly yielded similar results. Becker Scarpitta et al.^35^ observed higher thermophilisation rates of bryophyte compared to vascular plant communities in lowland forests in France over a period of ca. 35 years and Di Nuzzo et al.^81^ suggested higher responsiveness of bryophytes compared to vascular plants based on surveys along an elevational gradient in Mediterranean mountains.

The difference between bryophyte and vascular plant communities in the observed vs. expected shifts is relevant for the threat of extinction of species. On one hand, bryophyte species seem to successfully track favourable temperatures. But on the other hand, when the area with favourable temperatures is reduced, as is the case in high mountains because of the topography, they may faster be subjected to population decline, isolation and extinction. Moreover, bryophyte species which depend on substrates that are not formed fast enough at higher elevations such as thick humus layers (with e.g., *Plagiobryum demissum* (Hook.) Lindb.) are likely to be especially threatened to suffer (local) extinctions. Vascular plants, however, are in general much more dependent on the availability of soil than bryophytes. It is suggested that mountain vascular plants have substantial extinction debts, because of dispersal limitations and pronounced longevity^21,82,83^. Hence, they may maintain population sizes for a certain time, but may then go extinct due to competition or extreme climatic events when the distances to areas with suitable temperatures and favourable conditions for establishment exceed the dispersal capacities. We observed an increase of cryophilic vascular plant species in the alpine zone, indicating that (at least in the short term) they profit from climate warming. Most likely, because growth at high elevations is predominantly limited by abiotic constraints rather than by competition^84^. However, in the long-term competition is expected to decimate their numbers^24,82^ and it must be noted that the rather coarse grouping of species into three categories of temperature affinity in our study can hide within group replacements of species by more thermophilic ones.

Interestingly, although the number of meso- and thermophilic species generally increased for both lineages, we did not detect a decrease in the number of cryophilic species in any elevational zone. Such patterns have already been observed among vascular plants and have been assigned to an in-filling process. This process is suggested to result from invasions of species for which the habitat became suitable and transient persistence of species for whom the conditions became unsuitable or which in the long term will be ruled out by competition^6,24,82,85^. Lamprecht et al.^72^ specifically observed a slowdown of this in-filling process due to species losses on an Alpine summit, highlighting the threat to high alpine species from climate warming.

### Land use types

Our results show that land use plays a role for the magnitude of thermophilisation of plant communities. The NES was higher in managed grasslands compared to forests, but not significantly different in unmanaged open areas. This suggests that rare disturbances in forest alone cannot explain the decreased thermophilisation, because disturbance is low even in unmanaged open areas. In recent years, it has been repeatedly shown that the physiognomy of forests, compared to open areas, slows down climate warming effects due to a buffering effect of the microclimatic conditions in the interior of forests^18,66,86^. Furthermore, the trees represent barriers for dispersal^87^ and forests may therefore less easily be invaded by thermophilic species than open areas. Additionally, the effect of disturbance on thermophilisation is not necessarily unidirectional. Disturbance can promote the spread of individual cryophilic species to warmer regions^88^ and along elevational gradients it may shift species’ ranges up- as well as downslope^89^.

The non-significant difference between managed grasslands and unmanaged open areas, however, is probably also due to the distribution of study plots and elevational differences in management practices. Only few plots in unmanaged open areas were in the colline, montane and subalpine zone. And in the alpine zone, the management of grasslands is generally extensive due to low productivity^90^ and consequently accompanied by lower disturbances compared to managed grasslands at lower elevations.

### Life strategies

Unexpectedly, we did not detect an effect of life strategy on the NES of both, bryophyte and vascular plant communities. In the case of vascular plants, this could be related to the generally low representation of the short-lived strategy in the data set (27% of classified species, 13% of records of classified species; Supplementary Table S2 online). Bryophyte species with short-lived life strategy at least tended to respond faster than species with long-lived strategy (Supplementary Fig. S2 online) and we suggest that in fact, there is a difference between these two groups, but we could not corroborate this statistically. Significance is counteracted by high variances (Supplementary Fig. S2 online), which are probably related to the difficulties to sample bryophytes. Many species are easily overlooked because they are small and pioneer species (representing the short-lived strategy) may be restricted to the diaspora bank in individual surveys and are therefore not sampled. Higher responsiveness of short-lived life strategies to climate warming were observed across different taxonomic groups including vascular plants, small mammals and fish^11,53,91^ and is suggested to make species with short-lived life strategies more vulnerable to climate change, especially in the face of increased climate variability^92^. For example, populations of short-lived species that do not have persistent diaspores and that cannot reproduce for some years because of a series of unfavourable conditions may irrecoverably collapse. On the other hand, species’ potential for adaptive evolution increases with decreasing generation time^14,93^ but depends on adaptive capacity^94^ which is largely unknown in bryophytes.

## Conclusions

Response dynamics of plants to climate warming are heterogeneous; they differ between taxonomic groups as well as between land use types and along elevational gradients. Most notably, climate warming scenarios developed for vascular plants cannot be uncritically generalised to land plants as a whole because bryophytes react substantially faster, which has consequences for the threat of species. Overall, bryophytes seem better adapted to track favourable climatic conditions, but at the same time, high elevation species with limited possibilities to migrate upward may be at more immediate risk of (local) extinction. Increased thermophilisation of plant communities due to invasion with thermophilic and mesophilic species at high elevations corroborates the immediate vulnerability of mountain habitats to climate warming and the disequilibrium in time lags between major habitat types such as forests and grasslands needs to be considered when making predictions.

## Supplementary Information

Supplementary Information.

## Data Availability

Data and R Markdown documents are provided at https://github.com/TobiasRoth/moss-and-vascular-plants. Raw data for analyses are provided in the folder “data-raw” and the folder “R” contains the R-Script that was used to export the data from the BDM database and to do the analyses. The final version of the repository will be archived at Zenodo (https://zenodo.org/). Bryophyte specimens of all surveys are stored in Z + ZT. Voucher information is publicly available at the herbarium's web-page (https://www.herbarien.uzh.ch).
