# Supplementary material for: Thermophilisation of communities differs between land plant lineages, land use types and elevation
Source: Sci Rep. 2023 Jul 14;13:11395. doi: 10.1038/s41598-023-38195-6 (PMC10349125; doi:10.1038/s41598-023-38195-6)
Supplement: Supplementary file 1 — Supplementary Information. [file 41598_2023_38195_MOESM1_ESM.pdf]

Scientific Reports

Thermophilisation of communities differs between land plant lineages, land use types and elevation

Thomas Kiebacher*[1,2], Markus Meier[2], Tabea Kipfer[3] & Tobias Roth[3,4]

[1] State Museum of Natural History Stuttgart, Department of Botany, Rosenstein 1, 70191 Stuttgart, Germany

[2] University of Zurich, Department of Systematic and Evolutionary Botany, Zollikerstrasse 107, 8008 Zürich, Switzerland

[3] Hintermann & Weber AG, Austrasse 2a, 4153 Reinach, Switzerland

[4] Zoological Institute, University of Basel, Basel, Switzerland

* thomas.kiebacher@smns-bw.de

Supplementary Information

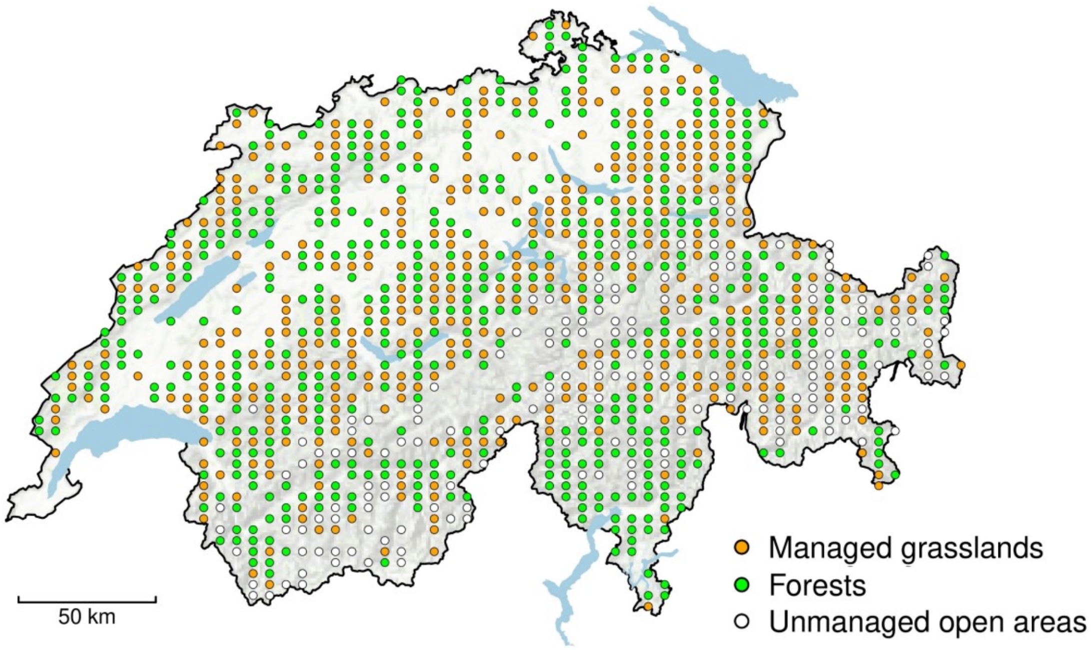

Figure S1. Distribution of the 1146 study plots across Switzerland. Every year one fifth of the plots is sampled and annual tranches are evenly distributed across major land use types, biogeographical regions and elevation. Assignment to the major land use type (managed grasslands, forests, unmanaged open areas) was based on a fine classification of 32 land use categories similar to the CORINE Land Cover system (Büttner et al. 2004) that are determined in the field at each survey. Background data source: Federal Office of Topography swisstopo.

Reference

Büttner G, Feranec J, Jaffrain G, et al (2004) The CORINE land cover 2000 project. EARSeL eProceedings 3:331–346.

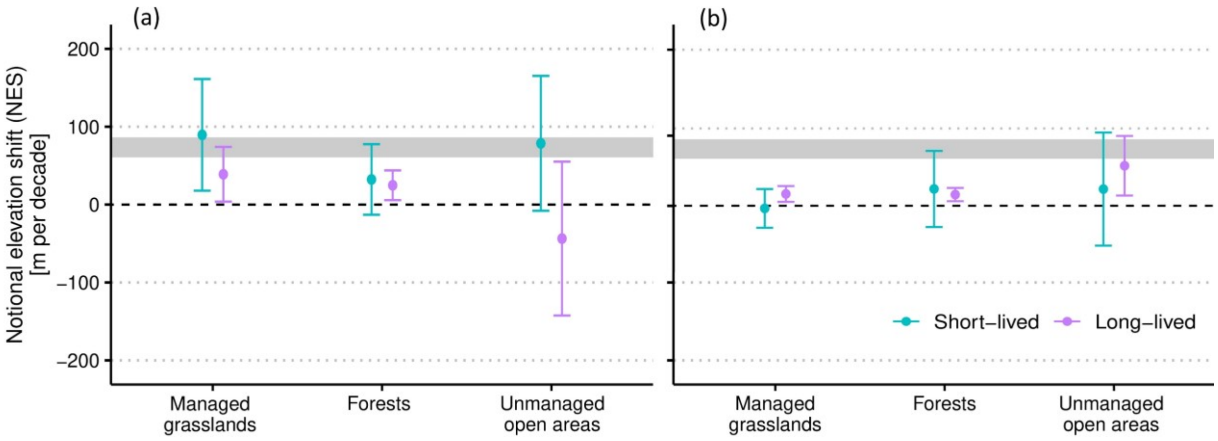

Figure S2. Mean NES of short and long-lived species in bryophyte (a) and vascular plant (b) communities in three major land use types. Points refer to the estimates of the life strategy and the whiskers are bootstrapped 95 % confidence intervals. The grey bar marks the observed upward shift of isotherms of 63 to 84 m per decade.

Table S1. List of recorded bryophyte species with Landolt (et al. 2010) indicator value for temperature (T) life strategy type (s = short-lived, l = long-lived) and number of records (N) in each elevational zone and in total. Species that can hardly be distinguished are fused to aggregate species (aggr.) according to Kiebacher et al. (in press), which were treated as species in the analyses. References: Kiebacher T, Meier M, Steffen J, Bergamini A, Schnyder N & Hofmann H (in press) Rote Liste der Moose. Swissbryophytes & Bafu, Zürich & Bern. Landolt E, Bäumler B, Erhardt A et al (2010) Flora Indicativa. Ecological indicator values and biological attributes of the flora of Switzerland and the Alps. Verlag Paul Haupt, Bern.

Species	T	Life strategy	N colline	N montane	N subalpine	N alpine	N total
Abietinella abietina	3	l	18	48	37	17	120
Amblyodon dealbatus	2	s	0	0	0	4	4
Amblystegium serpens	4	l	46	164	24	9	243
Amphidium mougeotii aggr.	1	s	3	1	0	2	6
Anastrophyllum assimile	3	l	0	1	0	0	1
Andreaea heinemannii	1	l	0	0	0	1	1
Andreaea nivalis	1	l	0	0	0	12	12
Andreaea rothii aggr.	1	l	0	0	4	0	4
Andreaea rupestris aggr.	1	l	3	9	14	54	80
Aneura pinguis		l	0	5	24	19	48
Anoectangium aestivum	2	s	0	2	0	0	2
Anomobryum julaceum aggr.	3	s	0	2	2	4	8
Anomodon longifolius	4	l	4	0	0	0	4
Anomodon viticulosus	4	l	23	40	0	0	63
Anthelia julacea aggr.	1	s	0	0	0	73	73
Antitrichia curtipendula	3	l	0	5	0	0	5
Asterella lindenbergiana	1	l	0	0	0	2	2
Atrichum angustatum	4	l	9	8	0	0	17
Atrichum undulatum aggr.	3	l	59	240	110	6	415
Aulacomnium palustre	3	l	0	3	11	24	38
Barbilophozia attenuata	3	s	0	1	16	2	19
Barbilophozia barbata	3	l	5	50	78	17	150
Barbilophozia floerkei	2	l	0	1	8	55	64
Barbilophozia kunzeana		s	0	0	1	5	6
Barbilophozia lycopodioides aggr.	2	l	0	44	198	394	636
Barbilophozia quadriloba	1	s	0	0	1	8	9
Barbula unguiculata	4	s	17	83	37	35	172
Bartramia halleriana	3	s	0	4	8	5	17
Bartramia ithyphylla	1	l	0	5	29	199	233
Bartramia pomiformis	3	l	1	1	0	0	2
Bazzania flaccida	3	s	1	7	0	1	9
Bazzania tricrenata	2	l	0	0	6	3	9
Bazzania trilobata	3	l	1	7	7	0	15
Blepharostoma trichophyllum	2	s	0	120	222	194	536

Blindia acuta aggr.	2	s	0	7	3	12	22
Blindiadelphus recurvatus	3	s	0	10	8	7	25
Brachydontium trichodes		s	0	0	2	0	2
Brachytheciastrum collinum		l	0	0	1	25	26
Brachytheciastrum trachypodium	1	l	0	0	0	4	4
Brachytheciastrum velutinum	3	l	74	557	312	131	1074
Brachythecium cirrosum aggr.	1	l	0	1	11	25	37
Brachythecium rivulare aggr.		s	218	1007	204	24	1453
Brachythecium salebrosum aggr.		s	26	242	344	233	845
Brachythecium sect. Albicans		l	0	8	11	11	30
Brachythecium tenuicaule aggr.	3	l	5	62	8	4	79
Bryoerythrophyllum ferruginascens	2	s	0	1	3	7	11
Bryoerythrophyllum recurvirostre aggr.		s	3	39	57	139	238
Bryoerythrophyllum rubrum	2	s	0	0	0	1	1
Bryum alpinum aggr.		l	0	1	0	6	7
Bryum archangelicum		l	0	0	1	3	4
Bryum argenteum	4	s	25	51	33	24	133
Bryum bicolor aggr.	4	s	5	12	5	2	24
Bryum blindii aggr.		l	0	0	0	1	1
Bryum caespiticium		l	0	1	1	2	4
Bryum capillare aggr.	3	l	85	369	286	300	1040
Bryum creberrimum	3	s	0	0	1	1	2
Bryum funckii		s	0	0	1	1	2
Bryum pallens aggr.		l	0	0	1	2	3
Bryum pallescens	2	s	0	1	2	6	9
Bryum pseudotriquetrum		l	2	15	43	52	112
Bryum subapiculatum aggr.	4	s	31	179	50	4	264
Bryum turbinatum aggr.	1	l	0	0	0	18	18
Bryum weigelii	1	l	0	0	5	3	8
Buckia vaucheri	1	l	0	1	2	4	7
Buxbaumia viridis	3	s	0	3	8	0	11
Calliergon giganteum		l	0	0	1	0	1
Calliergonella cuspidata	3	l	67	214	72	8	361
Calliergonella lindbergii	3	l	0	10	36	21	67
Calypogeia fissa	4	s	4	22	11	3	40
Calypogeia integristipula	2	s	0	8	30	18	56
Calypogeia muelleriana aggr.	3	s	1	30	94	42	167
Calypogeia neesiana	3	s	0	7	33	25	65
Calypogeia suecica	3	s	0	3	7	0	10
Campyliadelphus chrysophyllus		l	15	42	60	92	209
Campylium bambergeri	1	l	0	0	0	5	5
Campylium stellatum		l	14	58	166	160	398
Campylophyllopsis calcarea		l	11	28	15	22	76
Campylophyllum halleri	2	l	0	8	48	44	100
Campylopus atrovirens	4	l	0	0	1	2	3
Campylopus flexuosus	3	s	0	1	5	2	8
Campylopus fragilis	3	s	6	8	5	0	19

Campylopus gracilis		l	0	1	2	7	10
Campylopus introflexus	4	s	0	6	0	0	6
Campylopus pilifer		l	2	0	0	0	2
Campylopus pyriformis	3	s	1	2	2	3	8
Campylopus subulatus		s	0	4	1	10	15
Catoscopium nigritum	2	l	0	0	1	2	3
Cephalozia albescens	1	s	0	0	0	28	28
Cephalozia bicuspidata aggr.	2	s	1	30	96	83	210
Cephalozia catenulata	3	s	0	4	1	0	5
Cephalozia connivens	3	s	0	0	1	0	1
Cephalozia leucantha	2	s	0	0	1	0	1
Cephalozia lunulifolia		s	0	4	21	15	40
Cephalozia pleniceps	2	s	0	0	2	4	6
Cephaloziella divaricata		s	0	1	5	10	16
Cephaloziella phyllacantha	3	s	0	0	1	0	1
Cephaloziella rubella aggr.		s	0	1	0	1	2
Ceratodon purpureus aggr.		s	16	83	64	61	224
Chiloscyphus polyanthos aggr.	3	l	0	42	51	14	107
Cinclidotus fontinaloides	4	s	0	0	1	0	1
Cirriphyllum crassinervium	4	l	10	44	0	1	55
Cirriphyllum piliferum	3	l	8	199	187	18	412
Claopodium rostratum		l	0	4	0	0	4
Clevea hyalina	1	l	0	0	0	3	3
Climacium dendroides	3	l	8	50	45	8	111
Cololejeunea calcarea	3	l	0	12	4	2	18
Conocephalum conicum aggr.	3	l	0	12	21	6	39
Conostomum tetragonum	1	l	0	0	0	13	13
Coscinodon cribrosus aggr.	3	s	10	1	0	1	12
Cratoneuron filicinum aggr.		l	14	72	52	27	165
Ctenidium molluscum	3	l	17	384	233	110	744
Cynodontium gracilescens aggr.	2	s	0	1	2	0	3
Cynodontium polycarpon aggr.	3	s	0	11	16	8	35
Cyrtomnium hymenophylloides		l	0	0	4	9	13
Dichodontium pellucidum aggr.		s	1	24	55	75	155
Dicranella crispa		s	0	1	0	0	1
Dicranella grevilleana		s	0	0	1	8	9
Dicranella heteromalla	3	s	15	89	32	17	153
Dicranella schreberiana	3	s	1	7	4	1	13
Dicranella staphylina	4	s	10	30	1	0	41
Dicranella subulata	2	s	0	0	1	6	7
Dicranella varia aggr.	3	s	1	9	11	5	26
Dicranodontium denudatum	3	l	0	136	85	24	245
Dicranoweisia cirrata	4	s	1	0	0	0	1
Dicranum elongatum	1	l	0	0	0	3	3
Dicranum fuscescens aggr.	2	l	1	0	13	15	29
Dicranum majus	3	l	3	0	2	0	5
Dicranum montanum	3	l	48	198	182	31	459

Dicranum muehlenbeckii aggr.		l	0	1	19	127	147
Dicranum polysetum	3	l	0	10	4	0	14
Dicranum scoparium aggr.	3	l	16	371	475	203	1065
Dicranum spurium	3	l	0	0	0	1	1
Dicranum tauricum	2	l	3	14	16	0	33
Dicranum viride	4	l	3	2	0	0	5
Didymodon acutus aggr.	4	s	1	2	0	0	3
Didymodon fallax	3	s	2	18	13	30	63
Didymodon ferrugineus	3	s	0	20	15	12	47
Didymodon giganteus	3	l	1	0	6	5	12
Didymodon luridus		s	0	1	0	1	2
Didymodon rigidulus aggr.	3	s	1	27	13	12	53
Didymodon spadiceus	4	s	0	1	1	0	2
Didymodon vinealis aggr.	4	s	0	0	1	0	1
Diobelonella palustris	2	l	0	0	7	6	13
Diphyscium foliosum		s	7	12	7	42	68
Diplophyllum albicans		s	11	20	8	6	45
Diplophyllum obtusifolium aggr.		l	0	5	3	3	11
Diplophyllum taxifolium	1	s	1	6	27	88	122
Distichium capillaceum aggr.	1	s	4	18	56	244	322
Ditrichum flexicaule aggr.	3	s	2	23	72	195	292
Ditrichum heteromallum aggr.	1	s	0	1	8	37	46
Ditrichum lineare		s	0	0	0	2	2
Ditrichum pallidum	4	s	0	1	0	0	1
Ditrichum pusillum	3	s	0	0	1	0	1
Drepanium fastigiatum	2	l	0	0	0	3	3
Drepanocladus aduncus		l	3	0	1	0	4
Drepanocladus polygamus		l	1	0	0	0	1
Drepanocladus trifarius		l	2	0	0	2	4
Encalypta alpina	1	l	0	0	3	36	39
Encalypta ciliata	2	l	0	1	8	1	10
Encalypta microstoma		l	0	3	0	3	6
Encalypta rhaptocarpa aggr.		l	0	0	0	8	8
Encalypta streptocarpa	3	l	11	63	40	27	141
Encalypta vulgaris	1	l	0	1	2	16	19
Entodon concinnus	3	l	16	38	33	9	96
Ephemerum serratum	4	s	0	17	4	1	22
Eucladium verticillatum	4	l	0	2	3	2	7
Eurhynchiastrum pulchellum aggr.	1	l	6	15	15	45	81
Eurhynchium striatum aggr.	3	l	56	499	64	1	620
Fabronia ciliaris aggr.		l	1	0	0	0	1
Fabronia pusilla	5	l	1	0	0	0	1
Fissidens adianthoides aggr.	3	l	29	207	148	78	462
Fissidens bryoides aggr.	4	s	15	79	33	43	170
Fissidens celticus		s	0	1	1	0	2
Fissidens crassipes	4	l	0	0	1	0	1
Fissidens exilis	3	s	0	6	0	0	6

Fissidens osmundoides		s	0	1	1	19	21
Fissidens taxifolius	3	s	46	462	173	43	724
Fontinalis antipyretica	4	l	0	3	0	1	4
Fossombronia foveolata		s	0	2	0	0	2
Fossombronia pusilla aggr.		l	0	2	0	0	2
Frullania dilatata		l	92	218	13	0	323
Frullania fragilifolia	3	l	0	4	0	0	4
Frullania jackii	3	l	1	0	0	0	1
Frullania tamarisci	3	l	2	7	2	0	11
Funaria hygrometrica	4	s	9	11	1	2	23
Grimmia alpestris	1	l	1	0	6	36	43
Grimmia anodon		l	0	0	0	1	1
Grimmia caespiticia		s	0	3	0	8	11
Grimmia donniana aggr.	1	s	0	0	0	12	12
Grimmia elatior	3	s	1	0	2	0	3
Grimmia elongata		s	0	0	0	1	1
Grimmia funalis	1	s	0	1	0	2	3
Grimmia hartmanii aggr.		l	12	25	35	35	107
Grimmia incurva		s	0	0	0	3	3
Grimmia longirostris	2	l	3	4	0	0	7
Grimmia montana		s	1	0	0	0	1
Grimmia ovalis	3	s	1	3	1	0	5
Grimmia pulvinata	4	s	1	0	0	0	1
Grimmia ramondii	2	l	1	8	3	9	21
Grimmia sessitana		s	0	3	4	87	94
Grimmia tergestina aggr.		s	0	0	2	1	3
Grimmia torquata	2	l	0	1	0	0	1
Grimmia trichophylla aggr.		s	1	3	2	0	6
Gymnocolea inflata	2	s	0	0	0	11	11
Gymnomitrion alpinum		s	0	0	0	1	1
Gymnomitrion brevissimum	1	s	0	0	0	20	20
Gymnomitrion commutatum		s	0	0	0	4	4
Gymnomitrion concinnatum	1	s	0	2	0	70	72
Gymnomitrion corallioides	1	s	0	0	0	2	2
Gymnostomum aeruginosum aggr.	3	s	0	13	17	25	55
Gyroweisia tenuis	4	s	0	1	0	0	1
Habrodon perpusillus		l	0	0	1	0	1
Hamatocaulis vernicosus	3	l	3	0	0	0	3
Hedwigia ciliata aggr.	3	l	26	26	1	0	53
Herzogiella seligeri	3	l	25	226	78	0	329
Herzogiella striatella	2	l	0	1	4	10	15
Heterocladiella dimorpha	1	l	1	17	66	203	287
Heterocladium heteropterum aggr.		l	1	1	3	0	5
Homalia trichomanoides	4	l	18	36	0	0	54
Homalothecium lutescens	3	l	18	48	18	6	90
Homalothecium philippeanum	3	l	0	13	0	0	13
Homalothecium sericeum	4	l	31	35	7	0	73

Homomallium incurvatum	3	s	15	81	35	18	149
Hookeria lucens	3	l	0	15	6	0	21
Hydrogonium amplexifolium		s	0	0	1	0	1
Hydrogonium croceum	3	s	0	0	5	8	13
Hygroamblystegium tenax	4	l	0	4	2	0	6
Hygroamblystegium varium	4	l	9	8	5	0	22
Hygrohypnum luridum	3	l	0	18	23	8	49
Hylocomiadelphus triquetrus	3	l	19	227	373	84	703
Hylocomiastrum pyrenaicum	2	l	0	28	120	122	270
Hylocomiastrum umbratum	2	l	0	4	36	14	54
Hylocomium splendens	3	l	12	216	334	129	691
Hymenoloma crispulum aggr.	1	s	1	10	98	398	507
Hymenostylium recurvirostrum		s	0	2	3	5	10
Hypnum cupressiforme aggr.	3	l	258	1103	261	50	1672
Isopaches bicrenatus	3	s	0	0	0	1	1
Isopaches decolorans	1	s	0	0	0	9	9
Isopterygiopsis muelleriana	3	l	2	20	19	13	54
Isopterygiopsis pulchella	2	l	0	3	7	12	22
Isothecium alopecuroides	3	l	45	341	89	3	478
Isothecium myosuroides	3	l	0	7	0	0	7
Jochenia pallescens aggr.	3	l	0	0	4	0	4
Jungermannia atrovirens	3	s	0	6	6	1	13
Jungermannia pumila	3	s	0	0	0	1	1
Kiaeria blyttii		s	0	0	0	17	17
Kiaeria falcata	1	s	0	0	0	54	54
Kiaeria starkei	1	s	0	0	1	95	96
Kindbergia praelonga	4	l	0	14	2	0	16
Lejeunea cavifolia aggr.	3	l	6	50	5	2	63
Lepidozia reptans	3	s	0	83	89	15	187
Leptobryum pyriforme	3	s	1	1	0	0	2
Leptodictyum riparium	4	l	0	4	0	0	4
Leptodontium styriacum		s	0	0	1	0	1
Lescuraea incurvata	1	l	0	22	149	328	499
Lescuraea mutabilis aggr.	1	l	1	7	58	127	193
Lescuraea patens	1	l	2	1	5	25	33
Lescuraea plicata	2	s	2	12	66	117	197
Lescuraea radicosa		l	0	3	8	9	20
Leskea polycarpa	4	l	1	1	0	0	2
Leucobryum glaucum aggr.	4	l	10	14	15	30	69
Leucodon sciuroides	4	l	36	70	5	0	111
Lewinskya affinis aggr.	4	s	30	88	1	0	119
Lewinskya rupestris	3	s	5	5	0	0	10
Lewinskya speciosa aggr.		l	7	20	3	0	30
Lewinskya striata	3	l	20	60	10	0	90
Liochlaena lanceolata		s	0	1	1	0	2
Loeskeobryum brevirostre	4	l	4	3	1	0	8
Lophocolea bidentata	3	l	7	81	63	4	155

Lophocolea heterophylla aggr.	3	s	50	369	355	98	872
Lophozia ascendens		s	0	1	19	3	23
Lophozia sudetica	1	s	0	3	2	27	32
Lophozia ventricosa aggr.	2	s	0	8	52	59	119
Lophozia wenzelii	1	s	0	0	3	46	49
Lophoziopsis excisa	3	s	0	1	0	11	12
Marchantia polymorpha		s	2	0	1	4	7
Marchantia quadrata	2	l	0	2	29	38	69
Marsupella emarginata aggr.	2	s	0	3	0	1	4
Marsupella funckii	2	s	1	0	0	2	3
Marsupella sphacelata	1	s	0	0	0	1	1
Marsupella sprucei	1	s	0	1	0	2	3
Meesia uliginosa aggr.	1	l	0	0	0	36	36
Mesoptychia bantriensis aggr.	3	s	0	16	35	60	111
Mesoptychia heterocolpos	2	s	0	0	1	11	12
Metzgeria conjugata	4	l	5	10	1	0	16
Metzgeria furcata	3	l	78	370	55	1	504
Metzgeria pubescens	3	s	3	17	1	0	21
Metzgeria temperata		l	1	9	0	0	10
Metzgeria violacea		l	1	5	0	0	6
Microeurhynchium pumilum		l	0	0	1	0	1
Microhypnum sauteri		l	0	0	5	4	9
Microlejeunea ulicina	4	l	1	6	0	0	7
Mnium hornum	4	l	0	3	2	1	6
Mnium lycopodioides		l	1	2	2	1	6
Mnium marginatum	3	l	0	16	15	11	42
Mnium spinosum aggr.		l	0	90	177	88	355
Mnium stellare	3	l	5	25	27	25	82
Mnium thomsonii	3	l	0	15	31	27	73
Moerckia blyttii	1	l	0	0	1	25	26
Moerckia hibernica aggr.	3	l	0	0	0	1	1
Myurella julacea	1	s	0	5	15	47	67
Myurella tenerrima	2	s	0	0	0	6	6
Nardia geoscyphus	1	s	0	0	0	5	5
Nardia scalaris	1	s	0	3	3	25	31
Neckera besseri		l	1	3	0	0	4
Neckera complanata	4	l	32	77	3	0	112
Neckera crispa	3	l	5	48	7	0	60
Neckera pumila		l	1	6	0	0	7
Nogopterium gracile	3	l	1	0	0	0	1
Nowellia curvifolia	3	s	0	12	3	0	15
Nyholmiella obtusifolia	4	s	8	7	0	0	15
Obtusifolium obtusum	2	l	0	4	25	8	37
Odontoschisma elongatum		s	0	0	0	1	1
Odontoschisma fluitans	3	s	0	0	1	0	1
Oligotrichum hercynicum	1	s	0	0	2	29	31
Oncophorus virens aggr.	1	l	1	0	2	23	26

Orthothecium intricatum	3	l	0	9	43	77	129
Orthothecium rufescens	3	l	0	0	13	14	27
Orthotrichum anomalum	4	s	0	12	3	0	15
Orthotrichum diaphanum	4	s	9	8	0	0	17
Orthotrichum pallens		s	4	14	6	1	25
Orthotrichum patens		s	4	6	0	0	10
Orthotrichum pumilum aggr.	3	s	2	7	2	0	11
Orthotrichum rogeri		l	0	1	0	0	1
Orthotrichum scanicum		s	0	1	0	0	1
Orthotrichum stramineum	3	s	4	14	1	0	19
Oxyrrhynchium hians aggr.		s	147	661	126	9	943
Oxyrrhynchium schleicheri		l	0	4	3	0	7
Oxystegus tenuirostris aggr.	3	s	15	117	117	96	345
Palustriella commutata aggr.	3	l	2	19	33	109	163
Palustriella decipiens	2	l	0	2	23	14	39
Paraleucobryum enerve	1	l	0	1	3	38	42
Paraleucobryum longifolium aggr.	3	l	2	23	24	21	70
Pedinophyllum interruptum	3	l	0	14	11	4	29
Pellia endiviifolia	3	s	2	20	16	1	39
Pellia epiphylla aggr.	3	s	1	20	28	24	73
Peltolepis quadrata	1	l	0	0	1	4	5
Phascum cuspidatum	4	s	19	90	4	1	114
Philonotis calcarea	3	l	0	1	6	9	16
Philonotis fontana aggr.		l	0	0	10	10	20
Philonotis seriata	1	l	0	0	0	6	6
Physcomitrium pyriforme	4	s	6	4	0	0	10
Plagiobryum zieri	3	l	0	0	1	1	2
Plagiochila asplenioides aggr.	3	l	18	339	360	168	885
Plagiomnium affine aggr.	3	l	42	303	275	35	655
Plagiomnium cuspidatum	3	l	17	43	15	3	78
Plagiomnium rostratum	3	l	10	83	35	9	137
Plagiomnium undulatum	3	l	40	362	87	0	489
Plagiopus oederianus	3	l	0	2	16	1	19
Plagiothecium cavifolium aggr.	3	l	7	46	32	36	121
Plagiothecium denticulatum aggr.	3	l	5	159	238	100	502
Plagiothecium laetum aggr.	3	l	24	231	215	42	512
Plagiothecium nemorale		l	25	94	13	5	137
Plagiothecium platyphyllum		l	0	0	2	0	2
Plagiothecium undulatum	3	l	0	12	17	1	30
Plasteurhynchium striatulum	3	l	11	10	1	0	22
Platydictya jungermannioides	2	l	1	18	49	49	117
Platygyrium repens	4	l	58	81	6	0	145
Platyhypnum duriusculum	1	l	0	0	0	2	2
Platyhypnum molle		l	0	0	0	1	1
Pleuridium acuminatum		s	2	6	1	2	11
Pleuridium subulatum	4	s	0	9	3	0	12
Pleurochaete squarrosa	4	l	1	0	0	0	1

Pleurozium schreberi	2	l	4	62	185	88	339
Pohlia andalusica		s	0	0	1	14	15
Pohlia andrewsii		s	0	0	0	12	12
Pohlia annotina		s	0	0	2	4	6
Pohlia camptotrachela		s	0	0	3	0	3
Pohlia cruda	1	s	3	22	72	136	233
Pohlia drummondii		s	0	0	3	22	25
Pohlia elongata		s	0	2	3	5	10
Pohlia filum		s	0	0	1	19	20
Pohlia longicolla	2	s	0	0	0	1	1
Pohlia ludwigii	1	l	0	0	0	3	3
Pohlia lutescens	3	s	1	0	1	0	2
Pohlia melanodon	3	s	0	2	1	0	3
Pohlia nutans aggr.		s	0	2	28	32	62
Pohlia obtusifolia	1	s	0	0	0	2	2
Pohlia proligera		s	0	0	0	1	1
Pohlia wahlenbergii		l	2	8	45	52	107
Polytrichum alpinum	1	l	0	24	106	217	347
Polytrichum commune	3	l	2	4	19	32	57
Polytrichum formosum aggr.	3	l	34	282	205	52	573
Polytrichum juniperinum aggr.	1	l	0	17	58	289	364
Polytrichum longisetum	3	l	0	1	11	1	13
Polytrichum nanum aggr.	3	s	2	15	3	2	22
Polytrichum piliferum	1	l	3	4	26	290	323
Polytrichum sexangulare	1	l	0	0	0	107	107
Polytrichum urnigerum	2	s	2	8	30	85	125
Porella arboris-vitae	3	l	4	0	0	0	4
Porella cordaeana	2	l	0	3	1	0	4
Porella platyphylla aggr.		l	43	100	19	13	175
Pseudanomodon attenuatus	4	l	35	91	1	1	128
Pseudephemerum nitidum	3	s	0	2	0	0	2
Pseudoamblystegium subtile	3	s	4	22	2	0	28
Pseudocrossidium hornschuchianum	4	s	0	1	0	0	1
Pseudoleskeella catenulata	3	l	1	64	43	30	138
Pseudoleskeella nervosa	3	l	13	72	34	2	121
Pseudoleskeella rupestris		l	0	0	1	0	1
Pseudoleskeella tectorum		l	0	4	1	0	5
Pseudoscleropodium purum	3	l	24	98	13	2	137
Pseudostereodon procerrimus	1	l	0	0	1	5	6
Pseudotaxiphyllum elegans	3	s	20	63	20	12	115
Pterigynandrum filiforme	3	l	15	204	145	45	409
Ptilidium ciliare aggr.	3	l	0	28	62	13	103
Ptilium crista-castrensis	3	s	0	5	19	6	30
Pulvigera lyellii	3	l	35	93	4	0	132
Pylaisia polyantha	4	l	15	19	3	0	37
Racomitrium aciculare	2	s	0	1	0	0	1
Racomitrium aquaticum	3	s	12	6	2	2	22

Racomitrium canescens aggr.	1	s	8	9	48	257	322
Racomitrium fasciculare		l	0	3	0	2	5
Racomitrium heterostichum aggr.	1	s	2	18	25	156	201
Racomitrium lanuginosum	1	l	0	0	1	12	13
Racomitrium macounii		s	0	0	0	10	10
Racomitrium microcarpon		s	0	0	0	14	14
Radula complanata	3	l	115	501	242	125	983
Rhabdoweisia crenulata	3	s	0	3	0	1	4
Rhabdoweisia crispata	3	s	4	3	1	1	9
Rhabdoweisia fugax		s	9	22	20	22	73
Rhizomnium magnifolium		l	0	0	9	5	14
Rhizomnium pseudopunctatum	2	l	0	0	4	0	4
Rhizomnium punctatum	3	l	9	268	245	30	552
Rhodobryum roseum aggr.	3	s	5	29	69	14	117
Rhynchostegiella tenella	4	l	2	1	0	0	3
Rhynchostegium confertum	4	l	3	3	0	0	6
Rhynchostegium megapolitanum	4	l	4	2	0	0	6
Rhynchostegium murale	3	l	13	263	164	20	460
Rhynchostegium riparioides	4	l	1	8	0	0	9
Rhynchostegium rotundifolium	4	l	0	1	0	0	1
Rhytidiadelphus loreus	3	l	0	54	89	0	143
Rhytidiadelphus squarrosus aggr.	3	l	16	229	258	24	527
Rhytidium rugosum	3	l	7	14	20	25	66
Riccardia chamedryfolia		s	0	2	2	0	4
Riccardia latifrons	3	s	0	2	0	0	2
Riccardia multifida	3	s	0	14	18	0	32
Riccardia palmata	3	s	0	13	15	0	28
Roaldia revoluta aggr.	1	l	1	1	2	16	20
Sanionia uncinata	2	l	0	90	291	297	678
Sarmentypnum exannulatum	2	l	0	0	4	3	7
Sauteria alpina	2	l	0	0	0	1	1
Scapania aequiloba aggr.		l	0	24	77	63	164
Scapania curta	3	s	0	0	0	1	1
Scapania cuspiduligera		s	0	0	2	30	32
Scapania gymnostomophila		s	0	0	2	2	4
Scapania mucronata		s	0	0	1	0	1
Scapania nemorea	3	l	6	8	3	0	17
Scapania paludicola	2	s	0	0	2	0	2
Scapania scandica		s	0	0	1	0	1
Scapania uliginosa aggr.	2	l	0	0	0	4	4
Scapania umbrosa	3	s	0	0	8	1	9
Scapania undulata aggr.	2	l	0	0	3	1	4
Schistidium apocarpum aggr.		s	33	250	190	215	688
Schistidium flaccidum		s	0	0	0	1	1
Schistidium rivulare aggr.	1	l	0	0	0	12	12
Schistidium sordidum		s	0	0	0	4	4
Schistidium trichodon		s	0	4	11	4	19

Schistochilopsis incisa aggr.	1	s	0	1	14	40	55
Sciuro-Hypnum flotowianum		l	2	6	0	0	8
Sciuro-Hypnum plumosum	3	l	1	0	0	0	1
Sciuro-Hypnum populeum	3	l	69	211	72	7	359
Sciuro-Hypnum reflexum aggr.		l	14	85	313	328	740
Scorpidium revolvens aggr.	3	l	4	0	4	9	17
Scorpidium scorpioides	4	l	2	0	0	1	3
Seligeria donniana	3	s	0	1	0	2	3
Seligeria pusilla	3	s	0	1	2	1	4
Sematophyllum demissum	5	s	2	0	0	0	2
Serpoleskea confervoides	3	l	4	24	6	1	35
Solenostoma confertissimum	1	s	0	0	2	2	4
Solenostoma gracillimum	3	s	0	0	2	5	7
Solenostoma obovatum		s	0	0	0	6	6
Solenostoma sphaerocarpum	1	s	0	0	0	1	1
Sphagnum capillifolium aggr.	3	l	0	1	6	1	8
Sphagnum compactum	2	l	0	0	0	7	7
Sphagnum cuspidatum	3	l	0	0	1	0	1
Sphagnum denticulatum aggr.	3	l	0	1	0	0	1
Sphagnum girgensohnii	3	l	0	5	8	1	14
Sphagnum magellanicum aggr.	3	l	0	0	1	1	2
Sphagnum majus	2	l	0	0	3	0	3
Sphagnum palustre aggr.	4	l	0	0	1	1	2
Sphagnum papillosum	3	l	0	0	4	0	4
Sphagnum quinquefarium	3	l	0	3	11	0	14
Sphagnum recurvum aggr.	3	l	0	0	5	0	5
Sphagnum russowii	3	l	0	0	2	0	2
Sphagnum squarrosum	3	l	0	4	0	0	4
Sphagnum subsecundum	2	l	0	0	4	0	4
Sphagnum tenellum	3	l	0	0	1	0	1
Sphenolobus minutus	2	s	0	8	29	42	79
Splachnum ampullaceum		l	0	0	0	1	1
Splachnum sphaericum	2	l	0	0	0	2	2
Stegonia latifolia	1	s	0	0	0	4	4
Stereodon callichrous	2	l	0	2	15	5	22
Stereodon hamulosus		l	0	0	0	3	3
Stereodon pratensis	3	l	0	0	1	0	1
Straminergon stramineum	3	l	0	1	4	0	5
Streblotrichum convolutum	4	s	4	13	1	2	20
Syntrichia montana	4	s	0	1	0	0	1
Syntrichia papillosa	4	s	26	18	0	0	44
Syntrichia ruralis aggr.		s	14	78	72	283	447
Syntrichia virescens		s	3	0	0	1	4
Syzygiella autumnalis	3	s	0	2	2	0	4
Taxiphyllum wissgrillii	3	l	2	14	0	0	16
Tayloria acuminata		s	0	0	0	2	2
Tayloria froelichiana	1	l	0	0	0	14	14

Tayloria serrata aggr.	2	s	0	2	32	21	55
Tetraphis pellucida	3	s	7	140	114	11	272
Tetraplodon mnioides		l	0	0	0	1	1
Tetrodontium ovatum		s	0	0	1	0	1
Tetrodontium repandum		s	0	0	0	1	1
Thamnobryum alopecurum	4	l	6	3	0	0	9
Thamnobryum neckeroides		l	0	1	0	0	1
Thuidium recognitum aggr.		l	49	151	116	22	338
Thuidium tamariscinum	3	l	48	352	71	1	472
Timmia austriaca	2	l	0	0	6	15	21
Timmia bavarica	2	s	0	2	5	2	9
Timmia norvegica aggr.		s	0	0	0	3	3
Timmiella anomala aggr.		s	0	1	0	0	1
Tomentypnum nitens	3	l	0	0	0	1	1
Tortella fragilis	1	s	1	0	2	27	30
Tortella inclinata aggr.	4	s	2	2	7	36	47
Tortella tortuosa aggr.	3	l	26	363	301	358	1048
Tortula hoppeana	1	l	0	8	22	181	211
Tortula modica	4	s	0	1	0	0	1
Tortula mucronifolia		s	0	0	1	0	1
Tortula muralis	4	s	2	8	2	1	13
Tortula subulata aggr.		s	9	34	15	12	70
Tortula truncata	4	s	1	10	4	0	15
Trichodon cylindricus	3	s	2	19	14	6	41
Trichostomum brachydontium	3	l	0	2	2	2	6
Trichostomum crispulum	3	s	1	6	7	7	21
Trilophozia quinquedentata	2	l	0	13	18	29	60
Tritomaria exsecta	3	s	0	18	35	6	59
Tritomaria scitula	1	s	0	0	0	2	2
Ulota coarctata	3	l	1	1	0	0	2
Ulota crispa aggr.	4	l	44	142	14	0	200
Ulota hutchinsiae	3	s	1	0	0	0	1
Warnstorfia fluitans	3	l	0	0	1	0	1
Weissia brachycarpa	4	l	1	7	3	3	14
Weissia condensa	3	s	4	5	1	0	10
Weissia controversa	3	s	10	32	20	11	73
Weissia longifolia	4	l	3	22	4	0	29
Weissia rostellata		l	1	2	2	2	7
Weissia squarrosa		l	0	1	0	0	1
Weissia wimmeriana		s	0	1	2	0	3
Zygodon conoideus		s	0	1	0	0	1
Zygodon viridissimus aggr.	4	s	5	16	0	1	22

Table S2. List of recorded vascular plant species with Landolt (et al. 2010) indicator value for temperature (T) life strategy type (s = short-lived, l = long-lived) and number of records (N) in each elevational zone and in total. Species that can hardly be distinguished are fused to aggregate species (aggr.) which were treated as species in the analyses. A reference list of aggregate species is available at the GitHub-Repository https://github.com/TobiasRoth/moss-and-vascular-plants. To assign life strategy types to aggregate species not classified by Landolt et al. (2010), we used the category of the most common species of the aggregate based on occurrence data provided by Info Flora, the national data and information center on the Swiss flora (www.infoflora.ch). Reference: Landolt E, Bäumler B, Erhardt A et al (2010) Flora Indicativa. Ecological indicator values and biological attributes of the flora of Switzerland and the Alps. Verlag Paul Haupt, Bern.

Species	T	Life strategy	N colline	N montane	N subalpine	N alpine	N
Abies alba	3	l	59	612	108	0	779
Acer campestre	4	l	29	32	0	0	61
Acer opalus	4.5	l	12	1	0	0	13
Acer platanoides	4	l	16	65	2	0	83
Acer pseudoplatanus	3	l	134	698	158	3	993
Achillea atrata	1		0	0	0	58	58
Achillea erba-rotta	1		0	0	5	52	57
Achillea macrophylla	2	l	0	18	17	8	43
Achillea millefolium aggr.	3	l	42	269	160	11	482
Achillea nana	1		0	0	0	39	39
Achnatherum calamagrostis	3		0	7	4	0	11
Acinos alpinus	2		0	10	31	15	56
Acinos arvensis	4	s	3	0	2	1	6
Aconitum lycoctonum	2.5	l	4	24	49	6	83
Aconitum napellus	2.5	l	0	5	45	56	106
Aconitum variegatum	2	l	0	0	8	0	8
Actaea spicata	3	l	2	15	10	0	27
Adenostyles alliariae	2	l	0	16	139	41	196
Adenostyles alpina	2		0	61	60	12	133
Adenostyles leucophylla	1		0	0	0	5	5
Adoxa moschatellina	3		1	17	5	0	23
Aegopodium podagraria	3.5	l	18	86	6	0	110
Aesculus hippocastanum	4	l	0	1	0	0	1
Aethusa cynapium	3	s	0	2	0	0	2
Agrimonia eupatoria	4	l	11	14	0	0	25
Agrostis alpina	1.5		0	0	15	95	110
Agrostis canina	3.5		0	1	1	0	2

Agrostis capillaris	3		23	307	352	82	764
Agrostis gigantea	3.5	l	5	17	29	20	71
Agrostis rupestris	1		0	1	8	346	355
Agrostis schleicheri	2.5		0	0	1	1	2
Agrostis schraderiana	1.5		0	12	62	206	280
Agrostis stolonifera	3		110	363	106	32	611
Ailanthus altissima	4.5	s	2	0	0	0	2
Ajuga pyramidalis	2		0	4	38	29	71
Ajuga reptans	3.5		76	510	197	14	797
Alchemilla alpina bdm-agg.	2		0	30	139	139	308
Alchemilla pentaphyllea bdm-agg.	1		0	0	0	110	110
Alchemilla vulgaris bdm-agg.		l	8	495	381	236	1120
Alliaria petiolata	4	l	29	14	0	0	43
Allium carinatum	3.5		3	2	9	0	14
Allium lusitanicum	3		0	2	5	0	7
Allium oleraceum	3.5		6	9	1	0	16
Allium schoenoprasum	2.5		0	0	12	7	19
Allium sphaerocephalon	4.5		0	1	1	0	2
Allium ursinum	3.5		22	40	9	0	71
Allium victorialis	2		0	0	5	0	5
Allium vineale	4	s	1	2	0	0	3
Alnus glutinosa	4	l	0	2	0	0	2
Alnus incana	3	l	3	30	5	0	38
Alnus viridis	2	l	0	20	86	11	117
Alopecurus pratensis	3.5	l	32	232	26	0	290
Amaranthus blitum aggr.	4.5	s	2	4	0	0	6
Amaranthus graecizans	5	s	0	1	0	0	1
Amaranthus retroflexus	4	s	2	1	0	0	3
Amelanchier ovalis	3	l	4	9	0	0	13
Anagallis arvensis	4	s	1	1	0	0	2
Androsace alpina	1		0	0	0	30	30
Androsace chamaejasme	1.5		0	0	7	80	87
Androsace helvetica	1.5		0	0	0	1	1
Androsace obtusifolia	1.5		0	0	1	63	64
Androsace puberula	1.5		0	0	0	9	9
Androsace vitaliana	1.5		0	0	0	5	5
Anemone baldensis	1.5		0	0	0	6	6
Anemone narcissiflora	2		0	0	15	25	40
Anemone nemorosa	3.5		97	289	16	0	402
Anemone ranunculoides	4		1	0	0	0	1
Angelica sylvestris	3	l	5	51	4	0	60
Antennaria carpatica	1.5		0	0	0	21	21
Antennaria dioica	2		0	8	23	69	100
Anthericum liliago	4		7	7	2	0	16
Anthericum ramosum	3.5		5	5	0	0	10
Anthoxanthum odoratum aggr.			60	540	429	500	1529
Anthriscus nitida	2.5	l	0	2	4	0	6

Anthriscus sylvestris	3.5	l	10	107	15	1	133
Anthyllis vulneraria			4	23	64	159	250
Aphanes arvensis	4.5	s	0	1	0	0	1
Aposeris foetida	2.5	l	0	13	61	12	86
Aquilegia alpina	2		0	0	4	0	4
Aquilegia atrata	3		0	15	4	0	19
Aquilegia vulgaris	3		3	4	6	0	13
Arabidopsis thaliana	3.5	s	4	5	0	0	9
Arabis alpina	2		1	2	20	77	100
Arabis bellidifolia	1.5		0	0	0	21	21
Arabis caerulea	1		0	0	0	39	39
Arabis ciliata	2.5		4	14	35	20	73
Arabis hirsuta aggr.	3.5		8	10	11	1	30
Arabis nova	2.5	s	5	3	0	1	9
Arabis subcoriacea	2		0	0	0	16	16
Arabis turrita	3.5		6	2	0	0	8
Arctium lappa	3.5	l	0	3	0	0	3
Arctostaphylos alpina	1.5	l	0	0	5	0	5
Arctostaphylos uva-ursi	2	l	0	0	9	23	32
Arenaria biflora	1		0	0	0	58	58
Arenaria ciliata aggr.	1.5		0	0	2	52	54
Arenaria marschlinsii	1		0	0	0	1	1
Arenaria serpyllifolia aggr.	4	s	15	28	9	0	52
Arnica montana	2		0	3	52	158	213
Arrhenatherum elatius	4	l	76	235	11	4	326
Artemisia absinthium	4		4	0	0	0	4
Artemisia campestris	3.5		2	0	0	0	2
Artemisia genipi	1		0	0	0	16	16
Artemisia umbelliformis	1		0	0	4	7	11
Artemisia verlotiorum	4.5	l	1	1	0	0	2
Artemisia vulgaris	4		4	3	0	0	7
Arum maculatum	4		32	53	0	0	85
Aruncus dioicus	3.5	l	12	14	0	0	26
Asarum europaeum	4	l	6	16	2	0	24
Asperula cynanchica	3.5		4	4	11	0	19
Asperula taurina	4	l	0	16	1	0	17
Asplenium adiantum-nigrum	4		13	0	0	0	13
Asplenium ruta-muraria	2.5		4	12	1	0	17
Asplenium septentrionale	2.5		6	1	0	0	7
Asplenium trichomanes	3.5		29	29	9	0	67
Asplenium viride	2	s	0	9	34	38	81
Aster alpinus	2		0	0	7	30	37
Aster amellus	3.5		1	0	0	0	1
Aster bellidiastrum	2		0	51	194	197	442
Aster novi-belgii aggr.	4.5	l	3	0	0	0	3
Astragalus alpinus	1.5	l	0	0	4	19	23
Astragalus australis	1.5	l	0	0	4	4	8

Astragalus cicer	4	l	0	5	0	0	5
Astragalus frigidus	1.5	l	0	0	4	5	9
Astragalus glycyphyllos	3.5	l	2	6	2	1	11
Astragalus penduliflorus	2	l	0	0	6	4	10
Astrantia major	2.5	l	0	11	55	8	74
Astrantia minor	2.5	l	0	5	21	40	66
Athamanta cretensis	2		0	0	6	1	7
Athyrium distentifolium	2	l	0	2	28	49	79
Athyrium filix-femina	3	l	16	261	118	5	400
Atriplex patula	4	s	0	3	3	0	6
Atropa bella-donna	3.5		1	9	0	0	10
Avenella flexuosa	3		5	76	213	272	566
Barbarea intermedia	3	s	0	5	0	0	5
Barbarea vulgaris	3.5	s	0	1	3	0	4
Bartsia alpina	2		0	0	42	214	256
Bellis perennis	3.5		68	431	75	13	587
Berberis vulgaris	3	l	19	20	4	0	43
Betula pendula	3.5	l	21	46	9	0	76
Betula pubescens	3	l	1	1	1	0	3
Bidens frondosa	4.5	s	1	0	0	0	1
Biscutella laevigata			0	3	20	43	66
Blackstonia perfoliata	4	s	3	0	0	0	3
Blechnum spicant	2.5	l	0	10	51	3	64
Blysmus compressus	3		0	2	15	2	19
Botrychium lunaria	2		0	11	34	97	142
Brachypodium pinnatum	3	l	12	97	33	0	142
Brachypodium rupestre	3.5	l	3	23	7	0	33
Brachypodium sylvaticum	3.5		46	219	11	0	276
Brassica napus	4	l	0	4	0	0	4
Briza media	3		7	66	146	41	260
Bromus benekenii	3.5	l	1	35	5	0	41
Bromus erectus	4	l	35	106	19	1	161
Bromus hordeaceus	3.5	s	47	204	3	0	254
Bromus inermis	4.5	l	5	2	0	0	7
Bromus racemosus aggr.	4.5	s	0	5	0	0	5
Bromus ramosus	3.5	l	0	2	0	0	2
Bromus sterilis	4	s	11	4	0	0	15
Bromus tectorum	4.5	s	3	2	0	0	5
Buddleja davidii	4.5	l	0	3	0	0	3
Buglossoides arvensis	3.5	s	1	0	0	0	1
Buphthalmum salicifolium	3		0	5	4	0	9
Bupleurum falcatum	4		4	0	0	0	4
Bupleurum longifolium	3		0	0	1	0	1
Bupleurum ranunculoides	2		0	0	7	5	12
Bupleurum stellatum	1.5		0	0	8	17	25
Calamagrostis arundinacea	3	l	7	29	12	0	48
Calamagrostis canescens	3.5	l	1	0	0	0	1

Calamagrostis epigejos	3.5	l	3	3	1	0	7
Calamagrostis varia	3	l	0	102	102	27	231
Calamagrostis villosa	2	l	0	38	180	62	280
Calamintha nepeta aggr.	4		0	4	0	0	4
Calluna vulgaris	2.5		8	11	83	111	213
Caltha palustris	3		3	25	69	14	111
Calystegia sepium	3.5	l	12	3	0	0	15
Campanula barbata	2		0	10	90	198	298
Campanula cenisia	1		0	0	0	8	8
Campanula cochleariifolia	2		1	16	57	85	159
Campanula excisa	1.5		0	0	0	2	2
Campanula glomerata	4		4	2	7	0	13
Campanula patula	3.5		1	4	1	0	6
Campanula persicifolia	4		2	0	0	0	2
Campanula rapunculoides	4		3	22	4	0	29
Campanula rapunculus	4.5		0	3	0	0	3
Campanula rhomboidalis	2.5	l	0	45	39	1	85
Campanula rotundifolia	3.5		16	106	112	34	268
Campanula scheuchzeri	1.5		0	26	201	504	731
Campanula spicata	4		2	0	0	0	2
Campanula thyrsoides	2		0	0	1	3	4
Campanula trachelium	3.5	l	5	46	9	0	60
Capsella bursa-pastoris	3	s	46	134	8	0	188
Capsella rubella	4.5	s	4	5	0	0	9
Cardamine alpina	1		0	0	0	55	55
Cardamine amara	3		1	8	9	1	19
Cardamine flexuosa			11	55	23	0	89
Cardamine heptaphylla	3		0	61	11	2	74
Cardamine hirsuta	3.5	s	40	81	2	1	124
Cardamine impatiens	3.5		4	13	3	0	20
Cardamine kitaibelii	3.5		0	6	0	0	6
Cardamine pentaphyllos	3		0	15	6	0	21
Cardamine pratensis aggr.	3.5	l	65	391	37	0	493
Cardamine resedifolia	1.5		1	6	19	214	240
Cardamine trifolia	3	l	0	1	0	0	1
Cardaminopsis halleri	2.5		1	0	2	0	3
Carduus defloratus	2.5		0	44	109	87	240
Carduus nutans	4	s	0	0	1	0	1
Carduus personata	2.5	l	0	2	9	0	11
Carex acuta	4	l	2	0	0	0	2
Carex acutiformis	3.5	l	1	0	0	0	1
Carex alba	3		10	52	11	4	77
Carex atrata	1.5		0	0	0	23	23
Carex brizoides	4	l	7	19	1	0	27
Carex brunnescens	1.5		0	0	5	6	11
Carex canescens	2.5		0	0	5	2	7
Carex capillaris	1.5		0	0	3	40	43

Carex caryophyllea	3		8	37	52	20	117
Carex curvula	1	I	0	0	1	247	248
Carex davalliana	3		0	5	20	10	35
Carex digitata	3		40	116	24	3	183
Carex echinata	3		0	1	32	20	53
Carex elata	3.5		4	0	0	0	4
Carex ericetorum	3		0	0	3	21	24
Carex ferruginea	2	I	0	17	119	84	220
Carex firma	1.5		0	0	3	37	40
Carex flacca	3		21	241	146	37	445
Carex flava aggr.	3		8	6	39	23	76
Carex foetida	1		0	0	0	28	28
Carex frigida	1.5		0	1	9	29	39
Carex fritschii	4.5	I	0	1	0	0	1
Carex halleriana	4.5		10	0	0	0	10
Carex hartmanii	3.5		1	0	0	0	1
Carex hirta	3.5	I	17	83	14	0	114
Carex hostiana	3		1	0	2	0	3
Carex humilis	3.5		28	29	14	6	77
Carex lachenalii	1.5		0	0	0	5	5
Carex lasiocarpa	3		2	0	0	0	2
Carex leporina	3		0	19	45	13	77
Carex liparocarpos	4.5		1	0	0	0	1
Carex montana	3	I	20	114	93	6	233
Carex muricata aggr.	3.5	I	5	19	1	0	25
Carex nigra	2.5		6	10	74	34	124
Carex ornithopoda	3.5		9	142	124	79	354
Carex ornithopodioides	1		0	0	0	16	16
Carex pallescens	3		1	52	154	54	261
Carex panicea	3		8	20	47	9	84
Carex paniculata	3		1	4	12	5	22
Carex parviflora	1		0	0	0	65	65
Carex pauciflora	2		0	0	0	1	1
Carex pendula	3.5		8	30	0	0	38
Carex pilosa	4	I	12	7	0	0	19
Carex pilulifera	3.5		7	41	36	7	91
Carex pulicaris	3		0	0	2	0	2
Carex remota	3.5		9	36	4	0	49
Carex rostrata	3		0	0	6	0	6
Carex rupestris	1.5		0	0	0	5	5
Carex sempervirens	1.5		0	17	135	418	570
Carex strigosa	3.5		1	0	0	0	1
Carex sylvatica	3.5		46	391	127	13	577
Carex tomentosa	3.5		0	3	0	0	3
Carex umbrosa	4		1	0	1	0	2
Carlina acaulis	3		0	24	113	106	243
Carlina vulgaris aggr.	3		0	4	1	1	6

Carpinus betulus	4	l	34	19	0	0	53
Carum carvi	2.5	l	2	84	134	21	241
Castanea sativa	4.5	l	38	14	0	0	52
Caucalis platycarpos	5	s	2	0	0	0	2
Centaurea jacea			20	36	32	0	88
Centaurea montana	2.5	l	0	15	64	5	84
Centaurea nervosa	2	l	0	4	10	6	20
Centaurea nigrescens	4	l	9	0	4	0	13
Centaurea scabiosa		l	9	26	41	4	80
Centaurium erythraea	3.5	s	2	3	0	0	5
Cephalanthera damasonium	3.5		1	6	0	0	7
Cephalanthera longifolia	3.5		0	7	2	0	9
Cerastium alpinum	1.5		0	0	0	4	4
Cerastium arvense			4	21	51	92	168
Cerastium cerastoides	1		0	0	0	28	28
Cerastium fontanum	3.5		124	697	216	51	1088
Cerastium glomeratum	4	s	13	39	2	0	54
Cerastium latifolium	1		0	0	0	36	36
Cerastium ligusticum	5	s	0	1	0	0	1
Cerastium pedunculatum	1		0	0	0	22	22
Cerastium pumilum	4.5	s	0	1	0	0	1
Cerastium semidecandrum	4	s	1	0	0	0	1
Cerastium uniflorum	1		0	0	0	43	43
Chaenorrhinum minus	4	s	1	2	0	0	3
Chaerophyllum aureum	3	l	0	6	6	0	12
Chaerophyllum hirsutum aggr.	2.5	l	1	147	285	45	478
Chamorchis alpina	1.5		0	0	0	10	10
Chelidonium majus	3.5	l	3	1	0	0	4
Chenopodium album aggr.			8	27	1	0	36
Chenopodium bonus-henricus	2.5		0	12	3	6	21
Chenopodium hybridum	4.5	s	1	0	0	0	1
Chenopodium polyspermum	3.5	s	8	12	1	0	21
Chrysosplenium alternifolium	3		0	27	15	0	42
Cicerbita alpina	2	l	0	6	30	0	36
Circaea ×intermedia			0	2	0	0	2
Circaea alpina	2.5		0	22	7	0	29
Circaea lutetiana	3.5	l	37	107	3	0	147
Cirsium acaule	2.5		3	15	56	27	101
Cirsium arvense	3.5	l	1	40	5	0	46
Cirsium eriophorum	2.5	l	0	5	2	0	7
Cirsium erisithales	2.5	l	0	0	4	0	4
Cirsium helenioides	2.5	l	0	0	12	0	12
Cirsium oleraceum	3	l	2	66	15	1	84
Cirsium palustre	3.5		3	43	25	0	71
Cirsium rivulare	3.5	l	0	4	5	0	9
Cirsium spinosissimum	1.5	l	0	1	6	204	211
Cirsium vulgare	3.5	s	3	26	3	1	33

Clematis alpina	2	l	0	0	21	0	21
Clematis vitalba	3.5	l	35	38	0	0	73
Clinopodium vulgare	4	l	18	80	34	0	132
Coeloglossum viride	2		0	1	15	53	69
Colchicum alpinum	2.5		0	3	0	0	3
Colchicum autumnale	3		10	48	29	0	87
Colutea arborescens	4.5	l	0	1	0	0	1
Convallaria majalis	3.5	l	8	11	7	0	26
Convolvulus arvensis	4	s	6	21	0	0	27
Conyza canadensis bdm-agg.	4.5	s	14	4	0	0	18
Corallorhiza trifida	2.5		0	0	3	3	6
Cornus sanguinea	3.5	l	46	53	0	0	99
Coronilla vaginalis	2.5		0	4	2	0	6
Corydalis cava	3.5		1	0	0	0	1
Corydalis solida	3.5		0	4	0	0	4
Corylus avellana	3	l	74	239	25	0	338
Cotoneaster horizontalis bdm-agg.	4	l	2	0	0	0	2
Cotoneaster integerrimus	3	l	0	5	8	4	17
Cotoneaster tomentosus	3.5	l	2	2	1	0	5
Crataegus laevigata	3.5	l	16	10	1	0	27
Crataegus monogyna	4	l	28	43	2	0	73
Crepis alpestris	2	l	0	0	2	1	3
Crepis aurea	2	l	0	19	70	129	218
Crepis biennis	3.5	l	28	146	18	0	192
Crepis bocconei	2	l	0	0	0	1	1
Crepis capillaris	3.5	s	20	61	3	2	86
Crepis conyzifolia	2	l	0	6	10	7	23
Crepis kerneri	1.5		0	0	0	14	14
Crepis mollis	2.5		0	4	16	0	20
Crepis paludosa	3	l	0	40	73	4	117
Crepis pyrenaica	2.5	l	0	21	61	1	83
Crepis rhaetica	1		0	0	0	1	1
Crepis setosa	4.5	s	1	0	0	0	1
Crepis terglouensis	1		0	0	0	7	7
Crepis vesicaria	4	s	2	6	0	0	8
Crocus albiflorus	2.5		7	69	158	37	271
Cruciata glabra	4.5		4	4	0	0	8
Cruciata laevipes	3.5	l	0	9	12	0	21
Cryptogramma crispa	1.5		0	0	17	25	42
Cuscuta epithymum	3		0	5	13	0	18
Cuscuta europaea	4		0	1	0	0	1
Cyclamen purpurascens	4		5	11	1	0	17
Cynodon dactylon	5	l	1	0	0	0	1
Cynosurus cristatus	3		17	294	145	12	468
Cyperus fuscus	4	s	1	0	0	0	1
Cystopteris alpina	1.5		0	0	0	11	11
Cystopteris fragilis aggr.	3		1	21	18	22	62

Cystopteris montana	2		0	0	4	0	4
Cytisus nigricans	4.5	l	0	1	0	0	1
Cytisus scoparius	4	l	18	24	0	0	42
Dactylis glomerata	4		188	866	267	10	1331
Dactylorhiza incarnata			0	1	1	0	2
Dactylorhiza maculata	3		0	50	122	26	198
Dactylorhiza majalis	2.5		0	4	10	1	15
Danthonia decumbens	3		3	18	35	6	62
Daphne laureola	3.5	l	1	9	0	0	10
Daphne mezereum	3	l	0	11	28	10	49
Daphne striata	1.5	l	0	0	13	47	60
Daucus carota	4		25	60	6	1	92
Deschampsia cespitosa	3	l	17	107	246	153	523
Dianthus armeria	4.5	s	0	1	0	0	1
Dianthus carthusianorum			0	5	8	4	17
Dianthus superbus	3.5		0	3	20	4	27
Dianthus sylvestris	3		0	0	3	0	3
Digitalis grandiflora	2.5	l	0	1	3	0	4
Digitalis lutea	3.5	l	4	2	1	0	7
Digitaria ischaemum aggr.	4	s	2	5	0	0	7
Digitaria sanguinalis aggr.	4	s	18	14	0	0	32
Diphasiastrum alpinum	2		0	0	0	23	23
Doronicum clusii	1	l	0	0	0	45	45
Doronicum grandiflorum	1.5	l	0	0	3	37	40
Draba aizoides	1.5		0	1	0	51	52
Draba dubia	1		0	0	1	3	4
Draba siliquosa	1		0	0	0	3	3
Draba tomentosa	1		0	0	0	1	1
Drosera rotundifolia	3		0	0	2	0	2
Dryas octopetala	1.5		0	0	20	128	148
Dryopteris affinis	3	l	12	28	7	0	47
Dryopteris carthusiana	3	l	16	85	44	15	160
Dryopteris dilatata	2.5	l	8	188	116	40	352
Dryopteris expansa	2.5	l	0	4	9	3	16
Dryopteris filix-mas	3	l	13	213	83	8	317
Dryopteris villarii	2		0	0	1	4	5
Duchesnea indica	4.5	l	4	0	0	0	4
Echinochloa crus-galli	4	s	16	23	0	0	39
Echium vulgare	4	s	2	1	3	0	6
Eleocharis palustris aggr.	3		2	1	0	0	3
Eleocharis quinqueflora	3		1	0	2	4	7
Elymus caninus	3.5		3	48	13	0	64
Elymus repens	3.5	l	55	158	5	0	218
Elyna myosuroides	1.5	l	0	0	0	73	73
Empetrum nigrum	1.5		0	0	6	73	79
Epilobium alpestre	2.5	l	0	0	23	2	25
Epilobium alsinifolium	2	l	0	0	4	10	14

Epilobium anagallidifolium	1.5		0	0	2	28	30
Epilobium angustifolium	3	l	0	21	50	9	80
Epilobium ciliatum	4.5	s	0	1	0	0	1
Epilobium collinum	2.5		0	15	6	1	22
Epilobium dodonaei	4		0	0	0	1	1
Epilobium fleischeri	2		0	0	0	9	9
Epilobium hirsutum	3.5	l	1	3	0	0	4
Epilobium montanum	3		6	114	89	0	209
Epilobium nutans	2		0	0	1	10	11
Epilobium obscurum	3.5	l	1	5	0	0	6
Epilobium palustre	3		0	2	2	2	6
Epilobium parviflorum	3.5		7	15	2	0	24
Epilobium roseum	3.5		2	4	0	0	6
Epilobium tetragonum	3.5	l	1	13	0	0	14
Epipactis atrorubens	3		1	10	7	6	24
Epipactis helleborine aggr.	3		3	28	6	1	38
Epipactis palustris	3.5		0	1	0	0	1
Equisetum arvense	3.5	l	16	83	42	0	141
Equisetum fluviatile	3		1	0	0	0	1
Equisetum hyemale	3.5	l	0	5	0	0	5
Equisetum palustre	3	l	4	25	49	6	84
Equisetum sylvaticum	3	l	1	31	49	0	81
Equisetum telmateia	3.5		2	19	0	0	21
Equisetum variegatum	2.5		0	0	7	15	22
Erica carnea	2	l	0	8	34	50	92
Erigeron alpinus	1.5		0	0	2	17	19
Erigeron annuus	4	l	17	8	0	0	25
Erigeron glabratus	1.5		0	0	0	2	2
Erigeron neglectus	1		0	0	0	1	1
Erigeron uniflorus	1		0	0	1	43	44
Erinus alpinus	2.5		0	3	0	0	3
Eriophorum angustifolium	2.5		7	3	7	11	28
Eriophorum gracile	3.5		2	0	0	0	2
Eriophorum latifolium	3		0	1	9	2	12
Eriophorum vaginatum	2.5		0	0	4	1	5
Eritrichium nanum	1		0	0	0	6	6
Erophila verna aggr.	4	s	0	4	2	0	6
Erucastrum gallicum	4.5	s	0	1	0	0	1
Erucastrum nasturtiifolium	3.5		1	7	0	0	8
Erysimum rhaeticum aggr.	3		4	4	0	0	8
Euonymus europaeus	3.5	l	45	42	0	0	87
Euonymus latifolius	3.5	l	0	2	0	0	2
Eupatorium cannabinum	3.5	l	4	15	0	0	19
Euphorbia amygdaloides	4	l	5	28	0	0	33
Euphorbia cyparissias	3		22	43	75	24	164
Euphorbia dulcis	3.5		15	14	3	0	32
Euphorbia helioscopia	3.5	s	1	1	1	0	3

Euphorbia lathyris	5	l	1	0	0	0	1
Euphorbia peplus	4	s	1	2	0	0	3
Euphorbia platyphyllos	4	s	0	1	0	0	1
Euphorbia stricta	4		0	1	0	0	1
Euphorbia verrucosa	4.5		6	4	14	3	27
Euphrasia alpina	1.5	s	0	0	0	13	13
Euphrasia hirtella	2	s	0	0	6	4	10
Euphrasia minima	1.5	s	0	1	20	435	456
Euphrasia picta	2	s	0	0	1	2	3
Euphrasia rostkoviana			0	22	49	15	86
Euphrasia salisburgensis	2.5	s	0	1	19	36	56
Euphrasia stricta	3	s	0	0	1	4	5
Fagus sylvatica	3.5	l	114	624	53	0	791
Fallopia convolvulus	3.5	s	10	5	0	0	15
Fallopia dumetorum	3.5	s	2	0	0	0	2
Festuca altissima	2.5	l	0	41	6	0	47
Festuca arundinacea	3.5	l	29	86	6	1	122
Festuca gigantea	3.5		9	19	1	0	29
Festuca halleri bdm-agg.	1.5	l	0	0	6	267	273
Festuca heterophylla	4		10	4	0	0	14
Festuca ovina bdm-agg.			17	66	68	39	190
Festuca paniculata	3		0	0	4	0	4
Festuca pratensis	3		40	318	111	9	478
Festuca pulchella	2		0	0	10	21	31
Festuca quadriflora	1		0	0	16	148	164
Festuca rubra aggr.			90	576	488	279	1433
Festuca varia aggr.	2.5	l	10	49	14	27	100
Festuca violacea aggr.	1.5	l	0	0	15	130	145
Filipendula ulmaria	3	l	8	65	13	0	86
Filipendula vulgaris	3.5		1	0	0	0	1
Fourraea alpina	3		0	0	4	0	4
Fragaria vesca	3		43	375	195	4	617
Frangula alnus	3.5	l	10	9	0	0	19
Fraxinus excelsior	3.5	l	197	755	30	0	982
Fraxinus ornus	4.5	l	4	0	0	0	4
Fumana ericoides	5		1	0	0	0	1
Fumana procumbens	4.5		4	0	0	0	4
Fumaria officinalis	3.5	s	0	3	0	0	3
Gagea fragifera	2		0	0	0	1	1
Gagea lutea	3.5		0	1	0	1	2
Galega officinalis	4.5	l	1	0	0	0	1
Galeopsis ladanum	2.5	s	2	2	0	0	4
Galeopsis pubescens	4	s	2	0	0	0	2
Galeopsis tetrahit	3	s	59	226	71	3	359
Galinsoga quadriradiata	4	s	4	2	0	0	6
Galium anisophyllon	2	l	1	13	97	210	321
Galium aparine	3.5	s	46	73	3	0	122

Galium boreale	3		0	16	9	0	25
Galium lucidum	4.5		2	10	4	0	16
Galium megalospermum	1.5		0	0	0	28	28
Galium mollugo aggr.	3.5		59	249	38	4	350
Galium odoratum	3.5	l	53	367	48	1	469
Galium palustre	3		7	8	4	0	19
Galium pumilum	4		0	63	126	83	272
Galium rotundifolium	3	l	1	89	26	0	116
Galium rubrum	4		0	8	3	0	11
Galium sylvaticum	3.5	l	0	4	0	0	4
Galium uliginosum	3		5	6	1	0	12
Galium verum	4		4	22	14	0	40
Genista germanica	3.5	l	0	4	4	0	8
Genista sagittalis	3.5		5	0	4	0	9
Genista tinctoria	4.5	l	0	0	1	0	1
Gentiana acaulis	1.5		0	8	54	174	236
Gentiana asclepiadea	3	l	0	11	40	10	61
Gentiana bavarica	1.5		0	0	1	71	72
Gentiana brachyphylla	1		0	0	0	36	36
Gentiana campestris	2		0	0	27	103	130
Gentiana ciliata	3		0	0	6	5	11
Gentiana clusii	1.5		0	0	8	35	43
Gentiana germanica	3		0	2	1	3	6
Gentiana lutea	2.5	l	0	3	54	19	76
Gentiana nivalis	1.5		0	0	3	78	81
Gentiana orbicularis	1		0	0	0	22	22
Gentiana pneumonanthe	3.5		3	0	0	0	3
Gentiana punctata	1.5	l	0	0	2	66	68
Gentiana purpurea	2	l	0	0	29	65	94
Gentiana ramosa	1.5		0	0	1	26	27
Gentiana schleicheri	1		0	0	0	5	5
Gentiana tenella	1		0	0	0	12	12
Gentiana utriculosa	2.5		0	0	1	0	1
Gentiana verna	2.5		0	8	32	113	153
Geranium columbinum	4	s	1	9	0	0	10
Geranium dissectum	4	s	1	23	0	0	24
Geranium molle	4	s	6	4	0	0	10
Geranium palustre	4	l	0	1	0	0	1
Geranium pusillum	3.5	s	1	3	0	0	4
Geranium pyrenaicum	4	s	2	43	0	0	45
Geranium rivulare	2	l	0	0	0	1	1
Geranium robertianum	4		61	290	48	0	399
Geranium rotundifolium	4.5	s	1	0	0	0	1
Geranium sanguineum	3.5	l	1	4	0	0	5
Geranium sylvaticum	2.5	l	3	191	317	78	589
Geum montanum	1.5	l	0	4	45	292	341
Geum reptans	1	l	0	0	0	28	28

Geum rivale	3	l	0	65	43	1	109
Geum urbanum	3.5		62	122	12	0	196
Glechoma hederacea	4	l	119	285	4	0	408
Globularia bisnagarica	4.5		4	0	0	0	4
Globularia cordifolia	2.5		5	0	2	23	30
Globularia nudicaulis	2	l	0	2	24	29	55
Glyceria fluitans	3.5		0	2	2	0	4
Glyceria notata	3		2	2	6	0	10
Gnaphalium hoppeanum	1.5		0	0	0	12	12
Gnaphalium norvegicum	2		0	0	8	20	28
Gnaphalium supinum	1		0	0	1	263	264
Gnaphalium sylvaticum	3		0	0	10	11	21
Gnaphalium uliginosum	4	s	0	1	0	0	1
Goodyera repens	3		1	10	4	0	15
Gymnadenia conopsea	3		0	10	35	34	79
Gymnadenia odoratissima	3		0	4	0	2	6
Gymnocarpium dryopteris	2.5		0	37	86	7	130
Gymnocarpium robertianum	2.5		0	7	9	1	17
Gypsophila repens	2		2	1	25	38	66
Hedera helix	4	l	182	373	0	0	555
Hedysarum hedysaroides	1.5	l	0	0	16	66	82
Helianthemum alpestre	1.5	l	0	0	0	38	38
Helianthemum nummularium		l	10	24	110	103	247
Helictotrichon pratense		l	0	9	10	0	19
Helictotrichon pubescens	3		23	130	64	10	227
Helictotrichon versicolor	1.5		0	0	15	311	326
Helleborus foetidus	3.5	l	7	13	6	0	26
Hepatica nobilis	4		27	86	54	0	167
Heracleum sphondylium			28	273	66	5	372
Herniaria alpina	1		0	0	0	5	5
Hieracium alpicola	1		0	0	0	2	2
Hieracium alpinum	1.5		0	0	0	100	100
Hieracium amplexicaule	2.5		0	3	0	1	4
Hieracium angustifolium	1.5		0	0	2	47	49
Hieracium cymosum bdm-agg.	4		1	7	0	1	9
Hieracium intybaceum	2		0	0	6	23	29
Hieracium lactucella	3		3	45	122	53	223
Hieracium murorum bdm-agg.			44	325	477	147	993
Hieracium piliferum aggr.	1		0	0	0	50	50
Hieracium pilosella bdm-agg.			9	53	136	100	298
Hieracium pilosum	2		0	0	3	13	16
Hieracium prenanthoides aggr.	2	l	0	5	27	10	42
Hieracium staticifolium	2.5		0	0	4	9	13
Hieracium umbellatum bdm-agg.	4		5	10	1	0	16
Hieracium villosum	2		0	0	18	37	55
Hierochloë odorata	3	l	0	0	0	1	1
Hippocrepis comosa	3		4	32	82	68	186

Hippocrepis emerus	4	l	17	15	0	0	32
Holcus lanatus	3.5		101	454	16	0	571
Holcus mollis	3.5		3	24	0	0	27
Homogyne alpina	2		0	23	331	520	874
Hordelymus europaeus	3	l	0	84	28	0	112
Hordeum murinum	4	s	0	1	0	0	1
Humulus lupulus	4	l	1	4	0	0	5
Huperzia selago	2		0	21	35	72	128
Hypericum ×desetangsii	3.5		0	1	0	0	1
Hypericum hirsutum	4	l	1	21	0	0	22
Hypericum humifusum	3		0	9	0	0	9
Hypericum maculatum	2	l	0	21	109	35	165
Hypericum montanum	3.5		5	22	7	0	34
Hypericum perforatum	4		13	48	14	1	76
Hypericum tetrapterum	4	l	1	0	0	0	1
Hypochaeris radicata	4		10	117	44	4	175
Hypochaeris uniflora	2		0	0	8	26	34
Ilex aquifolium	3.5	l	10	22	0	0	32
Impatiens glandulifera	4	l	5	10	0	0	15
Impatiens noli-tangere	3.5	l	13	58	2	0	73
Impatiens parviflora	4	s	44	53	0	0	97
Iris pseudacorus	4		4	0	0	0	4
Jasione montana	4.5		1	2	0	0	3
Juglans regia	4	l	14	12	0	0	26
Juncus alpinoarticulatus	3.5		2	0	15	7	24
Juncus articulatus	3		3	22	29	6	60
Juncus bufonius	3.5	s	0	2	0	0	2
Juncus compressus	3		0	1	0	0	1
Juncus conglomeratus	3.5	l	3	3	0	0	6
Juncus effusus	3.5	l	7	97	55	5	164
Juncus filiformis	2.5		0	0	12	10	22
Juncus inflexus	3	l	0	9	1	0	10
Juncus jacquinii	1.5	l	0	0	0	71	71
Juncus tenuis	3.5		4	3	0	0	7
Juncus trifidus	1.5		0	0	12	104	116
Juncus triglumis	1.5		0	0	0	8	8
Juniperus communis		l	5	9	80	148	242
Kernera saxatilis	2.5		0	0	0	4	4
Knautia arvensis	3.5	l	27	47	11	0	85
Knautia dipsacifolia	3	l	0	106	174	19	299
Knautia drymeia	3.5	l	0	2	0	0	2
Kobresia simpliciuscula	1.5		0	0	0	1	1
Koeleria hirsuta	1.5		0	0	1	0	1
Koeleria pyramidata aggr.	3.5		5	9	19	4	37
Laburnum alpinum	4	l	1	4	0	0	5
Laburnum anagyroides	4.5	l	0	5	0	0	5
Lactuca perennis	4.5		2	3	0	0	5

Lactuca serriola	4.5	s	4	5	0	0	9
Lamium album	4	s	0	7	2	0	9
Lamium galeobdolon	3		68	425	111	0	604
Lamium maculatum	3.5		8	12	8	0	28
Lamium purpureum	3	s	16	45	0	0	61
Lappula deflexa	2.5	s	0	4	0	0	4
Lapsana communis	4	s	15	34	0	0	49
Larix decidua	2	l	0	40	74	16	130
Laserpitium gaudinii	2	l	0	4	9	0	13
Laserpitium halleri	2	l	0	0	26	29	55
Laserpitium latifolium	3	l	0	20	48	4	72
Laserpitium siler	3.5	l	0	4	3	0	7
Lathraea squamaria	4		1	0	0	0	1
Lathyrus heterophyllus	3.5	l	0	2	0	0	2
Lathyrus latifolius	4.5	l	0	1	0	0	1
Lathyrus linifolius	4		0	2	9	0	11
Lathyrus occidentalis	2	l	0	0	2	0	2
Lathyrus palustris	4		4	0	0	0	4
Lathyrus pratensis	3.5		21	264	45	0	330
Lathyrus sylvestris	3.5	l	0	3	0	0	3
Lathyrus vernus	3.5		11	64	8	0	83
Laurus nobilis	5	l	6	0	0	0	6
Leontodon autumnalis	3	s	4	54	97	34	189
Leontodon helveticus	1.5		0	0	49	481	530
Leontodon hispidus			33	126	216	263	638
Leontodon incanus	3		0	0	1	2	3
Leontodon montanus	1		0	0	1	28	29
Leontopodium alpinum	1.5	l	0	0	0	6	6
Lepidium virginicum	4.5	s	1	0	0	0	1
Leucanthemopsis alpina	1	l	0	0	1	355	356
Leucanthemum halleri	1.5		0	0	0	27	27
Leucanthemum vulgare aggr.			27	151	228	125	531
Leucojum vernum	3.5		1	0	2	0	3
Ligusticum mutellina	1.5	l	0	3	53	355	411
Ligusticum mutellinoides	1		0	0	0	77	77
Ligustrum vulgare	4	l	46	34	0	0	80
Lilium bulbiferum	3		0	6	2	0	8
Lilium martagon	3		0	7	18	6	31
Linaria alpina	1.5		0	0	4	76	80
Linnaea borealis	2		0	0	3	0	3
Linum alpinum	2		0	0	14	0	14
Linum catharticum	4		5	51	75	45	176
Listera cordata	2.5		0	0	11	2	13
Listera ovata	3		2	34	47	6	89
Lithospermum officinale	4	l	1	1	0	0	2
Lloydia serotina	1		0	0	4	17	21
Loiseleuria procumbens	1.5		0	0	0	121	121

Lolium multiflorum	4.5	l	87	281	5	1	374
Lolium perenne	3.5	l	157	689	69	0	915
Lonicera alpigena	2.5	l	0	17	33	0	50
Lonicera caerulea	2	l	0	0	0	1	1
Lonicera nigra	2.5	l	0	66	54	0	120
Lonicera periclymenum	4	l	0	4	0	0	4
Lonicera xylosteum	3.5	l	30	141	2	0	173
Lotus corniculatus aggr.			41	248	297	283	869
Lotus pedunculatus	3.5		1	12	3	0	16
Lunaria rediviva	3	l	0	4	4	0	8
Luzula alpinopilosa	1		0	1	14	265	280
Luzula campestris bdm-agg.			9	97	153	151	410
Luzula lutea	1.5		0	1	13	109	123
Luzula luzulina	2.5		0	24	75	5	104
Luzula luzuloides	3		4	19	19	2	44
Luzula nivea	2.5	l	30	99	47	0	176
Luzula pilosa	3		7	65	10	1	83
Luzula spicata	1.5		0	0	3	98	101
Luzula sylvatica aggr.		l	3	116	288	89	496
Lycopodium annotinum	2		0	9	45	4	58
Lycopus europaeus	4		4	0	0	0	4
Lysimachia nemorum	3	l	6	194	92	0	292
Lysimachia nummularia	3.5		37	63	2	1	103
Lysimachia vulgaris	3.5	l	8	7	0	0	15
Lythrum salicaria	4	l	12	5	0	0	17
Mahonia aquifolium	4.5	l	1	0	0	0	1
Maianthemum bifolium	3		5	104	89	4	202
Malus sylvestris	3.5	l	1	0	0	0	1
Malva moschata	4	l	0	1	0	0	1
Malva neglecta	3.5	s	0	2	0	0	2
Matricaria chamomilla	4	s	0	3	1	0	4
Matricaria discoidea	3.5	s	2	10	7	0	19
Medicago falcata	4	l	0	3	0	0	3
Medicago lupulina	4	s	28	152	28	0	208
Medicago sativa	4	l	5	10	0	0	15
Melampyrum pratense	3	s	6	17	13	13	49
Melampyrum sylvaticum	2.5	s	1	88	161	37	287
Melica ciliata	4.5		7	0	0	0	7
Melica nutans	3.5		13	98	42	1	154
Melica uniflora	4		6	9	0	0	15
Melilotus albus	4	s	1	0	0	0	1
Melilotus officinalis	4.5	s	0	9	0	0	9
Melittis melissophyllum	4		17	10	0	0	27
Mentha aquatica	3.5		8	1	0	0	9
Mentha arvensis	3.5		1	0	4	0	5
Mentha longifolia	3		1	20	5	0	26
Menyanthes trifoliata	3		1	0	4	0	5

Mercurialis perennis	3.5	l	39	279	29	3	350
Mespilus germanica	4.5	l	5	0	0	0	5
Milium effusum	3.5		4	16	12	0	32
Minuartia biflora	1		0	0	0	1	1
Minuartia laricifolia	2.5	l	0	3	0	0	3
Minuartia recurva	1.5		0	0	0	14	14
Minuartia sedoides	1		0	0	0	110	110
Minuartia verna	2		0	0	0	104	104
Minuartia viscosa	4.5	s	0	2	0	0	2
Moehringia ciliata	1		0	0	4	30	34
Moehringia muscosa	3		7	12	13	2	34
Moehringia trinervia	3		20	60	6	0	86
Molinia arundinacea	3		29	58	8	0	95
Molinia caerulea	3		8	13	48	8	77
Moneses uniflora	2.5		0	1	8	1	10
Monotropa hypopitys aggr.	3		0	2	2	0	4
Muscari comosum	4.5		0	4	0	0	4
Mycelis muralis	3.5		25	127	36	0	188
Myosotis alpestris	1.5		0	5	26	187	218
Myosotis arvensis	3.5	s	10	76	8	1	95
Myosotis decumbens	2		1	7	3	1	12
Myosotis nemorosa	3.5		0	15	4	0	19
Myosotis ramosissima	3.5	s	5	1	0	0	6
Myosotis scorpioides	3		1	21	24	0	46
Myosotis stricta	4	s	1	4	0	0	5
Myosotis sylvatica	3		2	48	34	1	85
Myosoton aquaticum	3.5	l	1	2	0	0	3
Myrrhis odorata	3	l	0	0	2	0	2
Narcissus ×verbanensis	3.5		0	2	0	0	2
Nardus stricta	2.5		0	31	189	357	577
Nasturtium officinale	4		0	1	0	0	1
Neottia nidus-avis	3.5		0	19	4	0	23
Nigritella rhellicani aggr.	1.5		0	0	6	48	54
Odontites luteus	4.5	s	1	0	0	0	1
Oenothera biennis aggr.	4.5	s	1	1	0	0	2
Onobrychis montana	2	l	0	0	21	0	21
Onobrychis viciifolia	4	l	4	13	1	0	18
Ononis natrix	4.5	l	3	0	0	0	3
Ononis repens	3.5	l	0	5	3	0	8
Ononis rotundifolia	4	l	1	0	0	0	1
Ophioglossum vulgatum	3		0	1	0	0	1
Oplismenus undulatifolius	5		4	0	0	0	4
Orchis mascula			0	3	1	1	5
Orchis morio	4		2	0	0	0	2
Oreochloa disticha	1		0	0	0	32	32
Oreopteris limbosperma	2.5		0	30	66	6	102
Origanum vulgare	3.5		15	20	6	0	41

Ornithogalum umbellatum aggr.			3	0	0	0	3
Orobanche caryophyllacea	4.5		0	2	0	0	2
Orobanche hederae	4.5		1	0	0	0	1
Orthilia secunda	2.5		2	20	27	0	49
Ostrya carpinifolia	4.5	l	5	2	0	0	7
Oxalis acetosella	3		42	645	339	29	1055
Oxalis stricta	4	s	12	7	0	0	19
Oxyria digyna	1		0	0	0	79	79
Oxytropis campestris	1.5	l	0	0	10	15	25
Oxytropis helvetica	1.5		0	0	0	3	3
Oxytropis jacquinii	1.5	l	0	0	0	15	15
Oxytropis lapponica	1.5	l	0	0	0	1	1
Panicum capillare aggr.	4.5	s	1	4	0	0	5
Papaver alpinum aggr.	1.5		0	0	0	5	5
Papaver rhoeas	4	s	0	4	0	0	4
Paradisea liliastrum	2	l	0	2	15	0	17
Paris quadrifolia	3		11	125	85	0	221
Parnassia palustris	2		3	6	68	91	168
Pastinaca sativa	4.5		0	3	0	0	3
Pedicularis ascendens	1.5		0	0	4	0	4
Pedicularis foliosa	2		0	1	4	4	9
Pedicularis kerneri	1		0	0	0	43	43
Pedicularis oederi	1.5		0	0	0	1	1
Pedicularis recutita	2		0	0	0	6	6
Pedicularis tuberosa	1.5		0	2	15	22	39
Pedicularis verticillata	1.5		0	0	7	54	61
Petasites albus	3	l	0	77	95	1	173
Petasites hybridus	3	l	0	4	3	0	7
Petasites paradoxus	2	l	1	4	11	1	17
Peucedanum carvifolia	4.5	l	0	2	0	0	2
Peucedanum cervaria	4	l	4	0	0	0	4
Peucedanum oreoselinum	4.5	l	1	2	8	0	11
Peucedanum ostruthium	2	l	0	8	70	44	122
Peucedanum palustre	4		6	0	0	0	6
Phalaris arundinacea	4	l	6	0	0	0	6
Phegopteris connectilis	3	l	1	35	37	2	75
Phleum alpinum aggr.	1.5	l	0	13	152	226	391
Phleum hirsutum	2	l	0	1	47	28	76
Phleum phleoides	4.5		4	1	3	0	8
Phleum pratense aggr.	3.5		43	295	46	1	385
Phragmites australis	4	l	16	0	0	0	16
Phyllitis scolopendrium	3		0	7	0	0	7
Phyteuma betonicifolium	2	l	20	61	127	70	278
Phyteuma globulariifolium	1		0	0	0	20	20
Phyteuma hedraianthifolium	1		0	0	0	1	1
Phyteuma hemisphaericum	1.5		0	0	7	264	271
Phyteuma orbiculare	2.5		0	31	92	50	173

Phyteuma ovatum	2	l	0	3	23	2	28
Phyteuma scheuchzeri	2.5		10	41	9	3	63
Phyteuma spicatum	3	l	22	171	131	9	333
Phytolacca americana	4.5	l	3	0	0	0	3
Picea abies	2.5	l	55	624	433	48	1160
Picris hieracioides	4	l	5	27	5	0	37
Pimpinella major	3	l	8	59	71	13	151
Pimpinella saxifraga aggr.	4		9	65	53	2	129
Pinguicula alpina	2		0	0	18	39	57
Pinguicula leptoceras	1.5		0	0	1	1	2
Pinguicula vulgaris	3		0	2	0	2	4
Pinus cembra	2	l	0	0	35	11	46
Pinus mugo	2	l	0	3	20	9	32
Pinus sylvestris		l	10	58	0	0	68
Plantago alpina	1.5		0	0	73	157	230
Plantago atrata	1.5		0	11	69	105	185
Plantago lanceolata	3		110	593	173	11	887
Plantago major	3		105	339	61	3	508
Plantago media	3.5		14	111	83	10	218
Plantago serpentina	2.5		0	0	5	2	7
Platanthera bifolia	3.5		1	9	2	0	12
Platanthera chlorantha	3.5		0	1	3	0	4
Poa alpina	1.5		0	13	138	521	672
Poa annua	3.5	s	89	262	43	2	396
Poa bulbosa	4.5		1	6	0	0	7
Poa cenisia	1.5		0	1	0	19	20
Poa chaixii	2.5	l	1	6	34	19	60
Poa compressa	4		4	2	1	0	7
Poa glauca	1.5		0	0	0	2	2
Poa hybrida	2.5		0	0	2	0	2
Poa laxa	1		0	0	0	146	146
Poa minor	1		0	0	0	59	59
Poa nemoralis	3		16	100	52	15	183
Poa palustris	4		1	0	0	0	1
Poa pratensis aggr.	3		115	536	89	2	742
Poa supina	2.5		1	29	36	21	87
Poa trivialis	3		172	687	125	1	985
Poa variegata	2		0	0	16	12	28
Polygala alpestris	2	l	0	4	68	66	138
Polygala alpina	1.5		0	0	1	5	6
Polygala amarella	3		2	9	3	3	17
Polygala chamaebuxus	2.5	l	3	54	97	55	209
Polygala pedemontana	4		0	0	2	0	2
Polygala serpyllifolia	3		0	0	1	1	2
Polygala vulgaris	3.5		1	13	23	4	41
Polygonatum multiflorum	3.5		40	42	2	0	84
Polygonatum odoratum	3		0	5	1	0	6

Polygonatum verticillatum	2.5		0	146	75	6	227
Polygonum aviculare aggr.	4	s	34	121	11	3	169
Polygonum bistorta	2.5	l	0	120	107	4	231
Polygonum hydropiper	3.5	s	1	2	0	0	3
Polygonum lapathifolium	3.5	s	1	0	0	0	1
Polygonum minus	4	s	0	2	0	0	2
Polygonum mite	4	s	3	0	0	0	3
Polygonum persicaria	3.5	s	4	6	0	0	10
Polygonum viviparum	1.5		0	6	95	399	500
Polypodium interjectum	4.5		0	1	0	0	1
Polypodium vulgare	3		12	51	7	0	70
Polystichum aculeatum	3	l	0	37	3	1	41
Polystichum lonchitis	2.5		0	2	28	21	51
Populus nigra aggr.			0	1	0	0	1
Populus tremula	3	l	11	28	1	0	40
Portulaca oleracea	4.5	s	4	0	0	0	4
Potentilla anserina	3		2	14	0	0	16
Potentilla argentea	3		0	5	0	0	5
Potentilla aurea	1.5	l	0	22	191	403	616
Potentilla brauneana	1		0	0	0	8	8
Potentilla crantzii	1.5	l	0	4	64	67	135
Potentilla erecta	3		5	127	312	96	540
Potentilla grandiflora	1.5	l	0	0	18	38	56
Potentilla inclinata	4.5		0	2	0	0	2
Potentilla micrantha	4.5		0	6	0	0	6
Potentilla pusilla	4		2	5	9	0	16
Potentilla recta	4.5		0	1	0	0	1
Potentilla reptans	3.5		44	114	0	0	158
Potentilla rupestris	3.5		3	2	0	0	5
Potentilla sterilis	3.5		13	114	4	0	131
Potentilla verna	4.5		6	15	15	3	39
Prenanthes purpurea	3	l	4	161	105	0	270
Primula acaulis	4.5	l	18	3	0	0	21
Primula auricula	1.5		0	0	8	13	21
Primula elatior	3	l	9	197	134	14	354
Primula farinosa	2		0	3	22	44	69
Primula hirsuta	1.5		0	9	7	76	92
Primula integrifolia	1.5		0	0	1	96	97
Primula latifolia	1		0	0	0	7	7
Primula veris			9	53	27	1	90
Pritzelago alpina	1		0	0	3	96	99
Prunella grandiflora	3.5		4	23	49	4	80
Prunella vulgaris	3		59	359	223	48	689
Prunus avium	4	l	58	98	0	0	156
Prunus domestica			3	3	0	0	6
Prunus laurocerasus	4.5	l	3	1	0	0	4
Prunus mahaleb	4.5	l	4	0	0	0	4

Prunus padus	3.5	l	4	24	0	0	28
Prunus serotina	4.5	l	2	0	0	0	2
Prunus spinosa	3.5	l	22	17	1	0	40
Pseudolysimachion spicatum	3.5		1	1	8	0	10
Pseudorchis albida	2		0	1	12	45	58
Pseudotsuga menziesii	3.5	l	0	5	0	0	5
Pteridium aquilinum	3	l	28	66	4	0	98
Pulicaria dysenterica	4	l	1	0	0	0	1
Pulmonaria australis	2	l	0	0	19	16	35
Pulmonaria mollis aggr.	3	l	0	5	3	0	8
Pulmonaria obscura	3.5	l	1	8	0	0	9
Pulmonaria officinalis aggr.	4.5	l	0	3	0	0	3
Pulsatilla alpina	2		0	1	33	87	121
Pulsatilla vernalis	2		0	0	10	71	81
Pyrola minor	2		0	0	4	17	21
Pyrola rotundifolia	2.5		0	1	2	1	4
Pyrus pyraster	4	l	1	1	0	0	2
Quercus cerris	4.5	l	1	0	0	0	1
Quercus petraea	4	l	23	25	0	0	48
Quercus pubescens	4.5	l	16	7	0	0	23
Quercus robur	4	l	36	51	3	0	90
Quercus rubra	4.5	l	5	2	0	0	7
Ranunculus aconitifolius	2.5	l	0	32	92	17	141
Ranunculus acris	3		134	741	224	20	1119
Ranunculus alpestris	1.5		0	0	3	114	117
Ranunculus auricomus aggr.	4		8	6	0	0	14
Ranunculus bulbosus	3		35	83	27	0	145
Ranunculus ficaria	3.5	s	102	310	10	0	422
Ranunculus flammula	3.5		4	1	6	0	11
Ranunculus glacialis	1		0	0	0	70	70
Ranunculus kuepferi	1.5		0	0	1	24	25
Ranunculus lanuginosus	2.5	l	0	19	39	0	58
Ranunculus montanus aggr.	2	l	0	48	235	355	638
Ranunculus parnassiifolius	1.5		0	0	0	1	1
Ranunculus platanifolius	2.5	l	0	7	14	1	22
Ranunculus repens	3	s	89	457	68	4	618
Ranunculus tuberosus aggr.	3	l	2	105	232	32	371
Reseda lutea	4.5		0	1	0	0	1
Reynoutria japonica aggr.	4	l	7	0	0	0	7
Rhamnus alpina	3.5	l	0	6	0	0	6
Rhamnus cathartica	3.5	l	0	1	0	0	1
Rhamnus pumila	2	l	0	1	0	0	1
Rhinanthus alectorolophus	3	s	13	55	62	6	136
Rhinanthus glacialis	2.5	s	0	1	19	35	55
Rhinanthus minor	3	s	0	9	21	10	40
Rhodiola rosea	1.5	l	0	0	3	4	7
Rhododendron ferrugineum	2	l	0	7	103	183	293

Rhododendron hirsutum	2	l	0	0	22	43	65
Rhus typhina	4.5	l	4	0	0	0	4
Ribes alpinum	2.5	l	0	12	7	0	19
Ribes petraeum	2.5	l	1	6	1	0	8
Ribes rubrum	3.5	l	0	3	0	0	3
Ribes uva-crispa	3.5	l	0	3	0	0	3
Robinia pseudoacacia	4.5	l	14	7	0	0	21
Rorippa ×anceps	4.5		1	0	0	0	1
Rorippa palustris	3.5	s	2	1	0	0	3
Rorippa sylvestris	3.5		0	1	0	0	1
Rosa arvensis	3.5	l	19	28	2	0	49
Rosa canina aggr.	3	l	5	24	1	0	30
Rosa corymbifera aggr.	3	l	0	1	0	0	1
Rosa multiflora	4.5	l	1	0	0	0	1
Rosa pendulina	2.5	l	0	30	90	5	125
Rosa rubiginosa aggr.	3.5	l	0	3	0	0	3
Rosa tomentosa aggr.		l	0	3	0	0	3
Rubus caesius	3.5	l	49	96	2	0	147
Rubus fruticosus s. l. bdm-agg.			134	439	14	0	587
Rubus idaeus	3		15	372	242	17	646
Rubus saxatilis	3		1	44	52	6	103
Rumex acetosa	3.5		63	482	100	1	646
Rumex acetosella	3.5		2	4	5	2	13
Rumex alpestris	2		0	32	128	30	190
Rumex alpinus	2	l	0	8	25	4	37
Rumex nivalis	1		0	0	0	23	23
Rumex obtusifolius	3.5	l	42	239	38	0	319
Rumex sanguineus	3.5	l	3	1	0	0	4
Rumex scutatus	2.5		10	11	23	10	54
Ruscus aculeatus	4.5	l	5	0	0	0	5
Sagina apetala	4	s	0	1	0	0	1
Sagina procumbens	3.5	s	2	11	7	2	22
Sagina saginoides	1.5		0	4	34	161	199
Salix alba	4	l	1	0	0	0	1
Salix appendiculata	2.5	l	1	17	48	6	72
Salix breviserrata	1.5	l	0	0	2	7	9
Salix caprea	3	l	3	24	18	3	48
Salix cinerea	3.5	l	4	3	4	0	11
Salix foetida	1.5	l	0	0	4	8	12
Salix glaucosericea	1.5	l	0	0	0	1	1
Salix hastata	2	l	0	0	4	5	9
Salix helvetica	1.5	l	0	0	0	17	17
Salix herbacea	1		0	0	2	259	261
Salix myrsinifolia	2.5	l	0	0	4	4	8
Salix purpurea	3	l	1	0	0	0	1
Salix reticulata	1.5		0	0	10	94	104
Salix retusa	1.5		0	0	13	165	178

Salix serpillifolia	1		0	0	0	33	33
Salix viminalis	3.5	l	0	4	0	0	4
Salix waldsteiniana	2	l	0	0	1	0	1
Salvia glutinosa	3	l	7	23	1	0	31
Salvia pratensis	3.5		13	21	10	0	44
Sambucus ebulus	4	l	0	4	0	0	4
Sambucus nigra	3.5	l	29	89	0	0	118
Sambucus racemosa	2.5	l	1	35	17	0	53
Sanguisorba minor	3.5		16	79	55	0	150
Sanguisorba officinalis	3.5	l	0	6	23	0	29
Sanicula europaea	3.5		0	46	14	0	60
Saponaria ocymoides	3		10	14	0	0	24
Saponaria officinalis	4.5	l	2	0	0	0	2
Saussurea alpina	1.5		0	0	0	10	10
Saussurea discolor	1.5		0	0	4	1	5
Saxifraga aizoides	2		0	1	23	144	168
Saxifraga androsacea	1		0	0	0	28	28
Saxifraga aphylla	1		0	0	0	9	9
Saxifraga aspera	2		0	3	0	7	10
Saxifraga biflora aggr.	1		0	0	0	30	30
Saxifraga bryoides	1		0	0	0	131	131
Saxifraga caesia	1.5		0	0	3	28	31
Saxifraga cotyledon	2.5		0	1	0	0	1
Saxifraga cuneifolia	2.5		1	16	31	0	48
Saxifraga exarata			0	1	2	76	79
Saxifraga muscoides	1		0	0	0	4	4
Saxifraga oppositifolia	1		0	0	8	147	155
Saxifraga paniculata	2		0	9	19	87	115
Saxifraga rotundifolia	2.5	l	0	21	88	15	124
Saxifraga seguieri	1		0	0	0	55	55
Saxifraga stellaris	1.5		0	4	10	86	100
Scabiosa columbaria	4		6	19	40	8	73
Scabiosa lucida	1.5		0	2	57	80	139
Scabiosa triandra	4.5		0	1	0	0	1
Scilla bifolia	4.5		7	0	0	0	7
Scirpus sylvaticus	3	l	0	18	5	0	23
Scleranthus annuus	3.5	s	0	1	0	0	1
Scrophularia nodosa	3	l	10	22	0	0	32
Scutellaria galericulata	3.5		2	0	0	0	2
Sedum acre	3		1	1	3	0	5
Sedum album	3		16	20	7	6	49
Sedum alpestre	1.5		0	2	1	177	180
Sedum anacampseros	1.5		0	0	0	3	3
Sedum annuum	2		0	2	2	3	7
Sedum atratum	1.5		0	0	0	58	58
Sedum cepaea	4.5	s	0	2	0	0	2
Sedum dasyphyllum	3.5		1	4	3	0	8

Sedum rupestre aggr.			4	8	0	2	14
Sedum sexangulare	3.5		8	10	0	0	18
Sedum telephium	3.5		15	4	0	0	19
Sedum villosum	2	s	0	0	0	2	2
Selaginella helvetica	3		0	5	3	0	8
Selaginella selaginoides	2		0	5	72	219	296
Sempervivum arachnoideum	2		0	9	1	27	37
Sempervivum montanum	1.5		1	4	20	206	231
Sempervivum tectorum			5	10	5	16	36
Senecio abrotanifolius	2		0	0	0	3	3
Senecio alpinus	2.5	l	0	7	16	5	28
Senecio doronicum	1.5		0	0	3	69	72
Senecio halleri	1		0	0	0	7	7
Senecio hercynicus	2	l	0	3	9	3	15
Senecio incanus	1		0	0	0	91	91
Senecio jacobaea	4	s	0	13	2	0	15
Senecio ovatus	3	l	3	37	35	10	85
Senecio paludosus	4		1	0	0	0	1
Senecio rupestris	2.5	s	0	0	2	0	2
Senecio sylvaticus	3.5	s	1	0	0	0	1
Senecio viscosus	2.5	s	3	2	0	0	5
Senecio vulgaris	3.5	s	11	3	0	0	14
Serratula tinctoria		l	2	0	0	0	2
Seseli libanotis	3	l	0	1	6	4	11
Sesleria caerulea	2	l	11	47	159	225	442
Setaria pumila	4	s	11	7	0	0	18
Setaria verticillata	5	s	2	0	0	0	2
Setaria viridis	4	s	3	5	0	0	8
Sibbaldia procumbens	1		0	0	3	140	143
Silene acaulis	1		0	0	12	137	149
Silene dioica	3		6	72	65	4	147
Silene exscapa	1		0	0	0	91	91
Silene flos-cuculi	3.5		5	29	6	0	40
Silene nutans	3	l	10	34	53	14	111
Silene pratensis	4		10	6	0	0	16
Silene pusilla	2.5		0	0	0	4	4
Silene rupestris	2.5		3	5	21	74	103
Silene vulgaris	3		18	73	105	71	267
Solanum dulcamara	3.5		1	10	0	0	11
Solanum nigrum	3.5	s	4	1	0	0	5
Soldanella alpina	2		0	11	166	291	468
Soldanella pusilla	1.5		0	0	8	191	199
Solidago canadensis	4	l	3	0	0	0	3
Solidago gigantea	3.5	l	0	1	0	0	1
Solidago virgaurea		l	19	189	282	218	708
Sonchus arvensis	3.5	s	1	6	0	0	7
Sonchus asper	3.5	s	6	43	3	0	52

Sonchus oleraceus	3.5	s	5	16	0	0	21
Sorbus aria	3.5	l	41	140	28	0	209
Sorbus aucuparia	3	l	7	340	324	25	696
Sorbus chamaemespilus	2	l	0	0	22	4	26
Sorbus mougeotii	3	l	0	1	4	0	5
Sorbus torminalis	4	l	0	4	0	0	4
Spiranthes spiralis	3.5		1	0	0	0	1
Stachys alpina	2.5	l	0	16	4	0	20
Stachys officinalis	3.5		16	30	11	0	57
Stachys palustris	3.5		2	0	0	0	2
Stachys recta	3.5		1	6	8	0	15
Stachys sylvatica	3.5		16	54	0	0	70
Stellaria alsine	2.5		0	12	4	0	16
Stellaria graminea	3.5		2	80	35	0	117
Stellaria media aggr.	3	s	85	261	21	0	367
Stellaria nemorum	2.5		1	37	63	5	106
Stipa capillata	3.5		1	0	0	0	1
Stipa pennata aggr.	3.5		0	0	0	1	1
Streptopus amplexifolius	2.5	l	0	8	23	3	34
Succisa pratensis	3		4	6	8	0	18
Swertia perennis	2.5		0	0	1	0	1
Symphytum asperum bdm-agg.	4.5	l	1	0	0	0	1
Symphytum officinale	3.5	l	8	6	0	0	14
Symphytum tuberosum	4	l	0	4	0	0	4
Tamus communis	4.5	l	11	2	0	0	13
Tanacetum corymbosum	4	l	0	1	0	0	1
Tanacetum vulgare	4.5	l	0	2	0	0	2
Taraxacum officinale bdm-agg.			210	924	252	180	1566
Taxus baccata	3.5	l	4	13	0	0	17
Teucrium chamaedrys	4.5		20	23	6	0	49
Teucrium montanum	3		4	2	2	0	8
Teucrium scorodonia	3.5		13	38	0	0	51
Thalictrum aquilegiifolium	2.5	l	0	6	23	5	34
Thalictrum minus			0	4	16	2	22
Thesium alpinum	2		0	7	63	121	191
Thesium pyrenaicum	2.5		0	2	4	4	10
Thlaspi alpestre aggr.	1.5		0	1	1	0	2
Thlaspi perfoliatum	4	s	5	10	1	0	16
Thlaspi rotundifolium	1.5		0	0	0	44	44
Thymus serpyllum aggr.			16	116	228	180	540
Tilia cordata	4	l	9	3	0	0	12
Tilia platyphyllos	4	l	10	31	0	0	41
Tofieldia calyculata	2.5		0	6	43	57	106
Torilis arvensis	4.5	s	1	0	0	0	1
Torilis japonica	4	s	0	5	0	0	5
Tozzia alpina	2.5		0	0	6	0	6
Trachycarpus fortunei	5	l	1	0	0	0	1

Tragopogon dubius	4.5		0	1	0	0	1
Tragopogon pratensis	4		6	62	29	0	97
Traunsteinera globosa	2		0	0	7	3	10
Trichophorum alpinum	3		0	0	0	1	1
Trichophorum cespitosum	2.5		0	0	12	15	27
Trifolium alpestre	4		0	5	0	0	5
Trifolium alpinum	1.5		0	0	19	167	186
Trifolium arvense	4	s	0	3	1	0	4
Trifolium aureum	3.5	s	0	1	3	0	4
Trifolium badium	2		0	5	61	127	193
Trifolium campestre	4	s	1	3	0	0	4
Trifolium dubium	3.5	s	6	46	1	0	53
Trifolium medium	3.5	l	6	58	44	0	108
Trifolium montanum	3	l	4	16	46	5	71
Trifolium ochroleucon	4	l	1	0	0	0	1
Trifolium pallescens	1.5		0	0	0	16	16
Trifolium pratense			102	645	347	159	1253
Trifolium repens	3		157	808	272	56	1293
Trifolium spadiceum	2.5		0	0	1	0	1
Trifolium thalii	1.5	l	0	1	21	62	84
Triglochin palustris	2.5		0	0	2	5	7
Tripleurospermum inodorum	3.5	s	0	1	0	0	1
Trisetum distichophyllum	1.5		0	0	6	20	26
Trisetum flavescens	3		46	344	86	5	481
Trisetum spicatum	1		0	0	0	28	28
Trollius europaeus	2.5	l	0	38	135	42	215
Tussilago farfara	3		3	37	76	27	143
Ulmus glabra	3.5	l	12	79	0	0	91
Urtica dioica	3.5	l	55	111	44	5	215
Vaccinium myrtillus	2.5	l	7	237	463	314	1021
Vaccinium uliginosum aggr.		l	0	1	55	272	328
Vaccinium vitis-idaea	2		0	43	231	187	461
Valeriana dioica	3		4	31	27	0	62
Valeriana montana	2		0	0	65	10	75
Valeriana officinalis aggr.			13	43	37	3	96
Valeriana supina	1		0	0	0	3	3
Valeriana tripteris	2.5		0	47	82	7	136
Valerianella carinata	4.5	s	2	1	0	0	3
Valerianella locusta	4	s	1	5	0	0	6
Veratrum album	2	l	0	22	147	61	230
Verbascum lychnitis	4		0	3	1	0	4
Verbascum thapsus	4		0	0	1	0	1
Verbena officinalis	3.5	s	6	5	1	0	12
Veronica agrestis	3.5	s	3	2	0	0	5
Veronica alpina	1.5		0	0	6	284	290
Veronica aphylla	1.5		0	0	4	59	63
Veronica arvensis	3.5	s	50	276	22	0	348

Veronica beccabunga	3		2	14	10	0	26
Veronica bellidioides	1		0	0	7	93	100
Veronica chamaedrys	3		62	584	302	17	965
Veronica filiformis	4		59	224	0	0	283
Veronica fruticans	1.5		0	7	8	68	83
Veronica fruticulosa	2		0	0	1	2	3
Veronica hederifolia	4	s	22	29	0	0	51
Veronica montana	3.5		13	35	4	0	52
Veronica officinalis	3	l	3	130	172	11	316
Veronica persica	3.5	s	47	115	1	0	163
Veronica serpyllifolia	3		61	409	108	5	583
Veronica teucrium	3.5	l	0	10	0	0	10
Veronica urticifolia	2.5	l	6	132	154	0	292
Veronica verna	4	s	1	3	0	0	4
Viburnum lantana	3.5	l	11	45	0	0	56
Viburnum opulus	3.5	l	2	34	0	0	36
Vicia cracca	3.5		8	65	27	0	100
Vicia hirsuta	4	s	2	3	0	0	5
Vicia onobrychioides	4.5	l	0	4	0	0	4
Vicia sativa	4.5	s	15	23	1	0	39
Vicia sepium	3	l	37	244	42	0	323
Vicia sylvatica	2.5	l	0	6	5	0	11
Vicia tetrasperma	4	s	1	0	0	0	1
Vinca minor	3.5	l	8	21	0	0	29
Vincetoxicum hirundinaria	3.5	l	21	9	6	2	38
Viola alba	4.5	l	2	4	0	0	6
Viola biflora	2		1	81	251	124	457
Viola calcarata	1.5	l	0	2	10	71	83
Viola canina	3		1	8	10	2	21
Viola cenisia	1		0	0	0	6	6
Viola collina	4	l	1	7	0	0	8
Viola hirta	3.5	l	8	41	18	2	69
Viola lutea	2	l	0	0	0	5	5
Viola odorata	4	l	0	1	0	0	1
Viola palustris	3		0	4	12	14	30
Viola pyrenaica	2.5	l	0	1	3	0	4
Viola reichenbachiana bdm-agg.	3	l	84	493	99	4	680
Viola rupestris	3		4	7	4	6	21
Viola thomasiana	2	l	0	6	16	6	28
Viola tricolor aggr.	3.5	s	6	22	9	1	38
Viscum album	4		1	3	0	0	4
Willemetia stipitata	2.5	l	0	1	19	8	28

Table S3. Data set to assess thermophilisation of plant communities and differences between lineages, land use types and elevation. Total number (N) of plots, mean elevation of plots, and number of plots with thermophilisation estimate for the bryophyte and vascular plant community, respectively; x̄NES: mean ± SD notional elevation shift.

Elevational zone	Land use type	N plots total	Mean Elevation [m a.s.l.]	Lineages			
				Bryophytes		Vascular plants	
				N plots	x̄NES	N plots	x̄NES
Colline	Managed grasslands	47	471	33	-79.4 ± 341.3	47	1.7 ± 56.3
Colline	Forests	67	548	67	63.4 ± 289.0	67	9.2 ± 97.4
Colline	Unmanaged open areas	2	367	2	-70.8 ± 107.1	2	168.1 ± 64.8
Montane	Managed grasslands	206	831	140	55.3 ± 551.9	206	5.0 + 46.6
Montane	Forests	304	949	304	13.6 ± 215.0	304	9.1 ± 83.6
Montane	Unmanaged open areas	2	1197	2	-139.2 ± 373.0	1	96.3 ± NA
Subalpine	Managed grasslands	88	1583	83	176.6 ± 889.1	88	23.5 ± 65.7
Subalpine	Forests	140	1599	140	23.5 ± 179.0	140	7.0 ± 61.9
Subalpine	Unmanaged open areas	13	1699	13	43.8 ± 273.4	13	37.9 ± 63.7
Alpine	Managed grasslands	115	2261	115	82.9 ± 269.2	115	18.8 ± 57.8
Alpine	Forests	12	1964	12	-61.7 ± 524.8	12	55.2 ± 124.9
Alpine	Unmanaged open areas	150	2391	146	81.4 ± 381.4	143	27.3 ± 87.1
Colline	All types	116	514	102	14.6 ± 310.2	116	8.9 ± 84.9
Montane	All types	512	903	446	26.0 ± 356.9	511	7.6 ± 70.9
Subalpine	All types	241	1598	236	78.5 ± 551.3	241	14.7 ± 63.9
Alpine	All types	277	2318	273	75.8 ± 346.3	270	25.0 ± 78.2
All zones	Managed grasslands	456	1300	371	79.0 ± 571.5	456	11.7 ± 55.1
All zones	Forests	523	1095	523	20.9 ± 228.4	523	9.6 ± 81.6
All zones	Unmanaged open areas	167	2299	163	73.9 ± 371.0	159	30.2 ± 86.1
All zones	All types	1146	1352	1057	49.5 ± 402.7	1138	13.3 ± 73.1

Table S4. Descriptive statistics of the data set. Mean number of species (N), mean community temperature index (CTI) and mean number of short-lived and long-lived species of bryophyte and vascular plant communities per survey across four elevational zones and three major land use types.

Elevational zone	Land use type	Bryophytes				Vascular plants			
		N total ± SD	CTI ± SD	N short-lived ± SD	N long-lived ± SD	N total ± SD	CTI ± SD	N short-lived ± SD	N long-lived ± SD
Colline	Forests	11.6 ± 5.9	3.24 ± 0.23	4.4 ± 3.1	19.9 ± 11.8	18.1 ± 9.8	3.45 ± 0.15	4.3 ± 6	31.1 ± 15.2
Colline	Managed grasslands	4 ± 3.2	3.48 ± 0.4	2.4 ± 3	4.6 ± 6.2	28.6 ± 10.7	3.43 ± 0.07	15.9 ± 9.6	22.2 ± 10.9
Colline	Unmanaged open areas	15.6 ± 3.7	2.83 ± 0.02	8.5 ± 6	15.5 ± 2.4	17.5 ± 10.6	3.4 ± 0.24	4.9 ± 7	22.8 ± 11.7
Montane	Forests	14.5 ± 7.3	3.06 ± 0.2	5.7 ± 4.2	26.6 ± 13.8	19.3 ± 11.8	3.15 ± 0.24	2 ± 3.7	29.5 ± 15.3
Montane	Managed grasslands	4.8 ± 5.3	3.42 ± 0.41	2.8 ± 3.1	5.3 ± 9.5	32.7 ± 11.9	3.32 ± 0.13	12.9 ± 8.4	22.9 ± 11.9
Montane	Unmanaged open areas	6.1 ± 5.5	2.97 ± 0.11	1.5 ± 2.1	7.1 ± 5.1	10.5 ± 14.5	3.46 ± 0.77	0.4 ± 0.5	13.9 ± 19.6
Subalpine	Forests	20.1 ± 8.4	2.72 ± 0.26	8.7 ± 5.7	31.9 ± 14.8	26.6 ± 12.2	2.63 ± 0.24	1.4 ± 1.6	29.1 ± 15.4
Subalpine	Managed grasslands	12.2 ± 8.6	2.91 ± 0.4	4.9 ± 4.5	14.4 ± 12.7	45.3 ± 13.7	2.75 ± 0.33	4.2 ± 4.5	28.9 ± 13.6
Subalpine	Unmanaged open areas	17 ± 11.5	2.73 ± 0.39	8 ± 6.3	19.5 ± 17	34 ± 18.1	2.39 ± 0.51	2.2 ± 2.2	23.9 ± 16.5
Alpine	Forests	19 ± 8.9	2.37 ± 0.46	8.4 ± 5.6	24.2 ± 13.8	20.9 ± 12	2.23 ± 0.23	0.8 ± 1	14.6 ± 7.2
Alpine	Managed grasslands	16.9 ± 8.1	1.95 ± 0.55	6 ± 4.3	12.4 ± 9.5	39.1 ± 13.3	1.78 ± 0.33	1.7 ± 1.2	15.3 ± 9.2
Alpine	Unmanaged open areas	14.1 ± 8.2	1.83 ± 0.6	5.4 ± 4.1	9.1 ± 8.8	20.9 ± 14.3	1.55 ± 0.37	0.8 ± 1	7.1 ± 8.2
All plots	–	12.9 ± 8.8	2.8 ± 0.68	5.3 ± 4.6	17.3 ± 15.3	27.3 ± 15.1	2.76 ± 0.71	4.5 ± 6.9	23.4 ± 15.2

Table S5. Results from the life strategy models. Effects of life strategy type, elevation, and land use type on the notional elevation shift (NES) of (a) bryophyte and (b) vascular plant communities

(a) Bryophytes.

**Predictor**	**Estimate**	**SE**	***p***
Notional elevation shift of short-lived bryophytes in grassland at 513 m a.s.l. (intercept)	62.10	29.22	0.034
Life strategy type: Long-lived	-21.92	16.72	0.190
Elevation (per 100 m)	0.01	1.79	0.997
Land use type: Forests	-24.94	22.22	0.262
Land use type: Unmanaged open areas	-37.31	27.49	0.175

(b) Vascular plants

**Predictor**	**Estimate**	**SE**	***P***
Notional elevation shift of short-lived vascular plants in grassland at 513 m a.s.l. (intercept)	3.55	11.08	0.749
Life strategy type: Long-lived	11.10	10.28	0.281
Elevation (per 100 m)	-0.44	0.87	0.608
Land use type: Forests	6.97	10.84	0.520
Land use type: Unmanaged open areas	35.08	18.38	0.057
